# Cost-effectiveness of intermittent preventive treatment with dihydroartemisinin–piperaquine for malaria during pregnancy: an analysis using efficacy results from Uganda and Kenya, and pooled data

**DOI:** 10.1016/S2214-109X(20)30369-7

**Published:** 2020-10-30

**Authors:** Silke Fernandes, Vincent Were, Julie Gutman, Grant Dorsey, Abel Kakuru, Meghna Desai, Simon Kariuki, Moses R Kamya, Feiko O ter Kuile, Kara Hanson

**Affiliations:** a Faculty of Public Health and Policy, Department of Global Health and Development, London School of Hygiene & Tropical Medicine, London, UK; b Kenya Medical Research Institute, Centre for Global Health Research, Kisumu, Kenya; c Infectious Diseases Research Collaboration, Kampala, Uganda; d Liverpool School of Tropical Medicine, Liverpool, UK; e Department of Medicine, University of California, San Francisco, CA, USA; f Malaria Branch, Division of Parasitic Diseases and Malaria, Center for Global Health, Centers for Disease Control and Prevention, Atlanta, GA, USA; g School of Medicine, Makerere University College of Health Sciences, Kampala, Uganda

## Abstract

**Background:**

Prevention of malaria infection during pregnancy in HIV-negative women currently relies on the use of long-lasting insecticidal nets together with intermittent preventive treatment in pregnancy with sulfadoxine–pyrimethamine (IPTp-SP). Increasing sulfadoxine–pyrimethamine resistance in Africa threatens current prevention of malaria during pregnancy. Thus, a replacement for IPTp-SP is urgently needed, especially for locations with high sulfadoxine–pyrimethamine resistance. Dihydroartemisinin–piperaquine is a promising candidate. We aimed to estimate the cost-effectiveness of intermittent preventive treatment in pregnancy with dihydroartemisinin–piperaquine (IPTp-DP) versus IPTp-SP to prevent clinical malaria infection (and its sequelae) during pregnancy.

**Methods:**

We did a cost-effectiveness analysis using meta-analysis and individual trial results from three clinical trials done in Kenya and Uganda. We calculated disability-adjusted life-years (DALYs) arising from stillbirths, neonatal death, low birthweight, mild and moderate maternal anaemia, and clinical malaria infection, associated with malaria during pregnancy. Cost estimates were obtained from data collected in observational studies, health-facility costings, and from international drug procurement databases. The cost-effectiveness analyses were done from a health-care provider perspective using a decision tree model with a lifetime horizon. Deterministic and probabilistic sensitivity analyses using appropriate parameter ranges and distributions were also done. Results are presented as the incremental cost per DALY averted and the likelihood that an intervention is cost-effective for different cost-effectiveness thresholds.

**Findings:**

Compared with three doses of sulfadoxine–pyrimethamine, three doses of dihydroartemisinin–piperaquine, delivered to a hypothetical cohort of 1000 pregnant women, averted 892 DALYs (95% credibility interval 274 to 1517) at an incremental cost of US$7051 (2653 to 13 038) generating an incremental cost-effectiveness ratio (ICER) of $8 (2 to 29) per DALY averted. Compared with monthly doses of sulfadoxine–pyrimethamine, monthly doses of dihydroartemisinin–piperaquine averted 534 DALYS (−141 to 1233) at a cost of $13 427 (4994 to 22 895), resulting in an ICER of $25 (−151 to 224) per DALY averted. Both results were highly robust to most or all variations in the deterministic sensitivity analysis.

**Interpretation:**

Our findings suggest that among HIV-negative pregnant women with high uptake of long-lasting insecticidal nets, IPTp-DP is cost-effective in areas with high malaria transmission and high sulfadoxine–pyrimethamine resistance. These data provide a comprehensive overview of the current evidence on the cost-effectiveness of IPTp-DP. Nevertheless, before a policy change is advocated, we recommend further research into the effectiveness and costs of different regimens of IPTp-DP in settings with different underlying sulfadoxine–pyrimethamine resistance.

**Funding:**

Malaria in Pregnancy Consortium, which is funded through a grant from the Bill & Melinda Gates Foundation to the Liverpool School of Hygiene and Tropical Medicine.

## Introduction

Malaria infection in pregnancy is associated with detrimental outcomes in the mother and the neonate, including foetal loss, low birthweight, maternal anaemia, and occasionally maternal or neonatal death.^1^, ^2^ In 2007, an estimated 30 million pregnancies occurred in the WHO African region in areas where *Plasmodium falciparum* is endemic.^3^ Without prevention, approximately 45% of livebirths in sub-Saharan Africa would be exposed in utero to *Plasmodium* infection, with most infections remaining asymptomatic in high transmission settings and resulting in adverse outcomes.^4^ Prevention of malaria infection in pregnancy in malaria-endemic settings is therefore crucial, and is a public health priority.

To prevent malaria infection, WHO currently recommends insecticidal nets combined with monthly intermittent preventive treatment in pregnancy with sulfadoxine–pyrimethamine (IPTp-SP) from the second trimester onwards in HIV-negative women, or daily co-trimoxazole prophylaxis in HIV-positive women.^5^ Despite high antenatal care attendance, coverage of IPTp-SP remains low in many settings.^2^, ^6^, ^7^ In addition, growing resistance of *Plasmodium* to sulfadoxine–pyrimethamine now threatens the effectiveness of intermittent preventive treatment for malaria in parts of sub-Saharan Africa. Thus it is crucial to identify an alternative to IPTp-SP.^8^

**Research in context**

**Evidence before this study**

Monthly administration of intermittent preventive treatment in pregnancy with sulfadoxine–pyrimethamine (IPTp-SP) is recommended by WHO to prevent malaria in HIV-negative women. Growing resistance to sulfadoxine–pyrimethamine threatens its effectiveness and alternative treatments are urgently needed. We did a literature review on prevention of malaria in pregnancy which was published in *The Lancet Infectious Diseases* in 2018. In addition, we searched PubMed on Sept 25, 2019, with the keywords “malaria” AND “pregnancy” AND “intermittent preventive treatment”, restricted to studies from 2017 onwards. Three trials of intermittent preventive treatment in pregnancy with dihydroartemisinin–piperaquine (IPTp-DP) were done in areas of high resistance to sulfadoxine–pyrimethamine, one in Kenya and two in Uganda. These trials compared IPTp-DP with IPTp-SP using different combinations of three doses or monthly administration of either drug combination. In all three trials, IPTp-DP was well tolerated, effective, and acceptable, suggesting that dihydroartemisinin–piperaquine is a promising preventive treatment candidate. However, to our knowledge, no cost-effectiveness analysis has been done to date.

**Added value of this study**

We estimated the cost-effectiveness of IPTp-DP regimens, using three different comparisons, in terms of prevention of clinical malaria and its sequelae from the health-care provider perspective using both individual trial results and, where possible, results pooled by meta-analysis. The cost-effectiveness comparing three doses of dihydroartemisinin–piperaquine with three doses of sulfadoxine–pyrimethamine using pooled data resulted in a highly cost-effective incremental cost-effectiveness ratio (ICER) of US$8 per disability-adjusted life year (DALY) averted. Similarly, the comparison of monthly doses of dihydroartemisinin–piperaquine (IPTp-DP_monthly_) versus monthly doses of sulfadoxine–pyrimethamine (IPTp-SP_monthly_), using data from the second Uganda trial, produced a highly cost-effective ICER of $25 per DALY averted. However, IPTp-DP_monthly_ was less effective and more costly than IPTp-DP3 and IPTp-SP3, driven by a small, non-significant increase in neonatal deaths in the IPTp-DP_monthly_ group of the first Uganda trial; a study with a small sample size of only 100 women per group. To address this, sensitivity scenarios modelling neonatal death were explored, generating highly cost-effective ICERs for IPTp-DP_monthly_ versus IPTp-DP3 and IPTp-SP3 ranging from $34 to $100 per DALY averted. For all three cost-effectiveness analyses, the likelihood of IPTp-DP being cost-effective, calculated using probabilistic sensitivity analysis, was high with respect to country-level cost-effectiveness thresholds for the base case scenario and in the latter analysis for the scenario in which neonatal death was modelled.

**Implications of all the available evidence**

Our results indicate that both IPTp-DP regimens are likely to be highly cost-effective at preventing malaria during pregnancy in settings of similar endemicity and sulfadoxine–pyrimethamine resistance. These results support earlier findings on the efficacy, safety, and acceptability of dihydroartemisinin–piperaquine, showing IPTp-DP could be a viable alternative strategy to prevent malaria during pregnancy in HIV-negative women.

Three recent clinical trials, one in western Kenya and two in eastern Uganda, found that intermittent preventive treatment in pregnancy with dihydroartemisinin–piperaquine (IPTp-DP) was effective in reducing clinical malaria in pregnancy and its sequelae, and was well tolerated when compared with IPTp-SP.^9^, ^10^, ^11^, ^12^ We refer to these trials as the Kenya,^12^ Uganda-I,^11^ and Uganda-II^10^ trials. Two of these trials compared a maximum of three or four doses of dihydroartemisinin–piperaquine (IPTp-DP3) versus three doses of sulfadoxine–pyrimethamine (IPTp-SP3). In the Uganda-I trial, the researchers included a third group in which women received a monthly dose of IPTp-DP (IPTp-DP_monthly_).^11^, ^12^ The Uganda-II trial compared IPTp-DP_monthly_ with monthly doses of IPTp-SP (IPTp-SP_monthly_; appendix p 2).^10^

We estimated the cost-effectiveness of these interventions in terms of their impact on preventing clinical malaria and its sequelae, summarised as disability-adjusted life-years (DALYs), in a decision tree model using the individual trial results and findings from the first two trials pooled by meta-analysis.^11^, ^12^ Cost-effectiveness analysis results presented here compare: IPTp-DP3 versus IPTp-SP3, using individual trial and pooled estimates from the Kenya trial and the Uganda-I trial;^11^, ^12^ IPTp-DP_monthly_ versus IPTp-DP3 and IPTp-SP3, using data from the Uganda-I trial;^11^ and finally IPTp-DP_monthly_ versus IPTp-SP_monthly_, using data from the Uganda-II trial.^10^

## Methods

### Study design and participants

We used outcome data from three clinical trials in Siaya County in western Kenya, and Tororo and Busia District in eastern Uganda, which enrolled HIV-negative pregnant women of all gravidities who had a high uptake of long-lasting insecticidal nets.^10^, ^11^, ^12^ To our knowledge, there are no other similar trials published. Although located in two different countries, the three trial sites are only a maximum of 110 km apart. The sites share a high intensity of malaria transmission and the effectiveness of IPTp-SP is compromised by widespread parasite resistance to sulfadoxine–pyrimethamine as a result of single-nucleotide polymorphisms in the *P falciparum* dihydrofolate reductase (*dhfr*) and dihydropteroate synthetase (*dhps*) genes. The prevalence of sulfadoxine–pyrimethamine quintuple-mutant parasites (a biomarker of resistance to sulfadoxine–pyrimethamine) is high, but the prevalence of the sextuple mutant is less than 6%.^13^ The Kenya trial randomly assigned 1031 women to receive either IPTp-SP3 (n=515) or IPTp-DP3 (n=516).^12^ A third group were assigned to receive intermittent screening followed by treatment with dihydroartemisinin–piperaquine, which was found to be unsuitable to replace IPTp-SP because of a lack of clinical efficacy, and this group was excluded from this cost-effectiveness analysis. The Uganda-I trial in Tororo, Uganda, randomly assigned 300 women to one of three groups, IPTp-SP3 (n=106), IPTp-DP3 (n=94), or IPTp-DP_monthly_ (n=100).^11^ The Uganda-II trial, in Busia District, Uganda, randomly assigned 782 women to either IPTp-DP_monthly_ (n=391) or IPTp-SP_monthly_ (n=391).^10^

Baseline characteristics were generally similar between the trials. In the Kenya trial, the gestational age of women at enrolment was 16–32 weeks (mean 22·8–23·0 weeks depending on group),^12^ while in the Uganda trials it was 12–20 weeks (mean 15·2–15·5 weeks in the Uganda-I trial^11^ and 15·0–15·4 weeks in the Uganda-II trial^10^). Women in the Uganda-I trial were on average younger than in the other two trials (mean 22·2 years *vs* 23·4 years in the Kenya trial and 23·0 years in the Uganda-II trial). Women in the Kenya trial received on average 2·7 doses of treatment in both groups; women in the Uganda-I trial received 2·8 (IPTp-SP3), 2·9 (IPTp-DP3), and 5·9 doses (IPTp-DP_monthly_); and women in the Uganda-II trial received 6·0 doses in both groups. The mean haemoglobin at baseline was lower in women in the Kenya trial compared with women in the Uganda trials (105 g/L in the Kenya trial *vs* 119 g/L in the Uganda-I trial and 115 g/L in the Uganda-II trial). Details of the study sites, trial design, participant characteristics, key outcomes, and sample size calculations have been previously published,^10^, ^11^, ^12^ and an overview is provided in the appendix (pp 3–4).

### Effects

Model outcomes were selected on the basis of clinical and economic relevance and availability of disability weights to calculate DALYs. Both child and maternal outcomes were included, namely neonatal death, stillbirth, low birthweight (<2·5 kg), maternal anaemia (haemoglobin <110 g/L for Kenya and Uganda-I and <100 g/L for Uganda-II), and clinical malaria. Separate decision tree models were developed for all child outcomes (neonatal death, stillbirth, and low birthweight), maternal anaemia, and clinical malaria (appendix pp 5–8), because disability weights for concurrent events (ie, maternal anaemia and clinical malaria) are unknown. In the decision tree for child outcomes, neonatal death branches off before low birthweight, as birthweight was not recorded for all babies and priority was given to the most severe outcome. Prevalence and incidence data in the model incorporated the individual trial results. Pooled effect estimates combining the effect of IPTp-DP3 versus IPTp-SP3 from the Kenya and Uganda-I trials were obtained using fixed-effects meta-analyses. DALYs were calculated by adding up the DALYs from all outcomes included in the model. Other serious adverse events and tolerance measures documented in the trial were excluded from the model (for both effect and cost), as no study drug-related difference in their incidence was recorded.

All three trials concluded that IPTp-DP was efficacious; however, the main outcomes driving their conclusions differed among the trials. Both the Kenya trial and the Uganda-II trial found a lower number of stillbirths and neonatal deaths in the IPTp-DP group, and the Uganda-I trial identified no difference in stillbirths, but a small, non-significant increase in neonatal deaths, probably occurring by chance because of the small sample size of the trial. The number of low birthweight babies in the IPTp-DP group increased in the Kenya trial, but was lower in both of the Uganda trials. Only the Uganda-II trial found a decrease in maternal anaemia in the IPTp-DP group, and in the other trials the level of anaemia was relatively similar between IPTp-SP and ITPp-DP groups. Finally, in all three trials, episodes of clinical malaria were substantially lower in all IPTp-DP groups.

### Costs

We adopted a health-care provider perspective to estimate the incremental fixed and variable costs of delivering the interventions and their cost savings. The costs of nurses' time, drugs, and of treating consequential health outcomes of malaria infection in pregnancy, comprising maternal anaemia, maternal clinical malaria, and post-delivery hospitalisation costs for low birthweight, were included. Health-care provider costs of a neonatal death or stillbirth (such as mortuary costs) were not collected because we did not anticipate these outcomes would drive the trials results, and hence these costs are excluded from the cost estimation. Cost data were collected by doing an observational study of trial participants in Kenya to measure the average administration time for intermittent preventive treatment (n=44) which was multiplied by the mean cost of nurses' time to estimate the total cost of nurses' time per administration; health-care facility costings (n=4) to estimate the costs of malaria infection during pregnancy and its sequelae; and an analysis of international drug procurement databases to calculate the drug prices per administration.^14^ The cost of nurses' time was estimated by averaging salaries and benefits provided to nurses obtained from Ministries of Health in Kenya and Uganda for 2018. For all costs, economic costs were calculated and valued in constant 2018 US$ using the local consumer price index and average 2018 exchange rates.^15^, ^16^ No health-care facility costing was done in Uganda. Therefore, the costs of malaria infection during pregnancy (and its consequences) from Kenya were used for Uganda, adjusting costs by the ratio of average nurse salaries in the two countries, which was 0·30 (ie, nurses' salaries in Uganda are 30% of those in Kenya). Ex-factory drug prices from procurement databases were adjusted for insurance and freight (10%), in-country transport (10%), and wastage (5%). Assumptions used in calculating costs of maternal anaemia and malaria infection during pregnancy are published elsewhere.^14^

All cost and observational data collection was approved by the ethics committees of the London School of Hygiene & Tropical Medicine, US Centers for Disease Control and Prevention, and the Kenya Medical Research Institute. Verbal consent was obtained from each participant being observed.

### Statistical analysis

Outcome data for IPTp-SP3 and IPTp-DP3 from the Uganda-I and Kenya trials were pooled using both fixed-effects and random-effects meta-analysis models, with the effect of the random-effects model explored in the sensitivity analysis. Because we followed the structure of the decision trees, the results are slightly different from the results reported by Desai and colleagues^8^ in a review and meta-analysis. The cost-effectiveness of IPTp-DP3 versus IPTp-SP3 (pooled estimate); IPTp-DP_monthly_ versus IPTp-DP3 and IPTp-SP3 (Uganda-I); and IPTp-DP_monthly_ versus IPTp-SP_monthly_ (Uganda-II), was analysed based on the decision trees from the health-care provider perspective using a lifetime horizon to reflect the lifelong mortality and morbidity effects of the adverse health outcomes of malaria infection during pregnancy. DALYs were calculated and then summed for all child and maternal outcomes using disability weights from Global Burden of Disease studies,^17^, ^18^, ^19^ case fatality rates from secondary literature (except for low birthweight, which is captured in the mortality estimates of the trials),^20^, ^21^ and local life expectancies, with no age weighting, and a discount rate of 3%.^22^

The results were expressed as an incremental cost-effectiveness ratio (ICER) for a hypothetical cohort of 1000 pregnant women, calculated by dividing the incremental costs of the intervention by the DALYs averted—ie,

$$\text{ICER}=\frac{\left({\text{Costs}}_{\text{IPTp}-\text{DP}3}-{\text{Costs}}_{\text{IPTp}-\text{SP}3}\right)}{\left({\text{DALY}}_{\text{IPTp}-\text{SP}3}-{\text{DALY}}_{\text{IPTp}-\text{DP}3}\right)}$$

The robustness of our results was tested using deterministic and probabilistic sensitivity analysis. The probabilistic sensitivity analysis included 10 000 iterations, producing a point estimate for each iteration (a simulation point), which was plotted in the cost-effectiveness plane. Subsequently, using all 10 000 iterations, the mean, median, and credibility intervals (CrIs; a 95% CI based on percentiles) of the differences in costs, effects, and ICER were calculated. Appropriate distributions were assigned to each parameter following cost-effectiveness guidelines (Table 1, Table 2).^22^ For zero events (neonatal death in IPTp-SP3 group and clinical malaria in the IPTp-DP_monthly_ group in the Uganda-I trial), a transformation of 0 plus 0·1 was used in the probabilistic sensitivity analysis to be able to model uncertainty of these parameters using distributions. The probabilistic sensitivity analysis results were compared with multiple country-specific cost-effectiveness thresholds and, for each threshold, the likelihood of being cost-effective was calculated. The cost-effectiveness thresholds were calculated by using estimates of country-level thresholds by Woods and colleagues^27^ and Ochalek and colleagues,^28^ adjusted for inflation. These thresholds ranged from $79·8 to $1273·1 for Kenya and from $30·6 to $793·0 for Uganda. Cost-effectiveness thresholds should be used as a guide for interpreting the results, rather than as an actual cutoff for cost-effectiveness. The Uganda-I trial was relatively small compared to the other trials (300 participants *vs* 1031 participants in the Kenya trial and 782 participants in the Uganda-II trial). The Uganda-I trial reported one stillbirth in each group, and zero neonatal deaths in 98 participants in the IPTp-SP3 group, two (2%) in 88 participants in the IPTp-DP3 group, and three (3%) in 97 participants in the IPTp-DP_monthly_ group. All five neonatal deaths in the Uganda-I trial occurred in the first 3 days of life and the causes of death were prematurity related complications. These differences in number of neonatal deaths were not significant; however, when these numbers are extrapolated to a hypothetical cohort of 1000 women in our models, the IPTp-DP_monthly_ group can never be more cost-effective than the other treatment regimens, because of the higher number of neonatal deaths in this group. The numbers of stillbirths and neonatal deaths in the three groups in the Uganda-I trial are very small; therefore the differences among the groups cannot be estimated precisely and might have occurred by chance (table 2). Therefore, although we used the trial results in the base case analysis, we also modelled mortality in the deterministic sensitivity analysis for the cost-effectiveness analysis of IPTp-DP_monthly_ versus IPTp-DP3 and IPTp-SP3.

**Table 1:** Cost and DALY input parameters

	**Base case**	**Distribution**	**Source**
**Health-care worker time costs**			
Time nurses take to provide one dose of preventive treatment in Kenya, s (95% CI)	377·16 (254·28–500·04)	Gamma	Observational study of trial participants, Kenya (n=44)
Mean number of doses per woman in the IPTp-SP3 group (Kenya)	2·74	Point estimate	Desai et al (2015)^12^
Mean number of doses per woman in the IPTp-DP3 group (Kenya)	2·71	Point estimate	Desai et al (2015)^12^
Mean number of doses per woman in the IPTp-SP3 group (Uganda-I)	2·82	Point estimate	Kakuru et al (2016)^11^
Mean number of doses per woman in the IPTp-DP3 group (Uganda-I)	2·85	Point estimate	Kakuru et al (2016)^11^
Mean number of doses per woman in the IPTp-DP_monthly_ group (Uganda-I)	5·94	Point estimate	Kakuru et al (2016)^11^
Mean number of doses per woman in the IPTp-SP_monthly_ group (Uganda-II)	6·00	Point estimate	Kajubi et al (2019)^10^
Mean number of doses per woman in the IPTp-DP_monthly_ group (Uganda-II)	6·00	Point estimate	Kajubi et al (2019)^10^
Nurses' monthly cost of labour in Kenya, US$ (range)	1037·86 (1002·84–1072·88)	Gamma	Ministry of Health, Kenya (2018)
Nurses' monthly cost of labour in Uganda, $ (range)	306·30 (219·71–392·89)	Gamma	Ministry of Health, Uganda (2018)
**Drug costs**			
International sulfadoxine–pyrimethamine price per tablet, $ (95% CI)	0·07 (0·05–0·08)	Gamma	International drug price list (2015) adjusted for inflation and Global Fund procurement database (2018)^23^, ^24^
International dihydroartemisinin–piperaquine price per tablet, $ (95% CI)	0·25 (0·22–0·29)	Gamma	International drug price list (2015) adjusted for inflation and Global Fund procurement database (2018)^23^, ^24^
Mean number of tablets per dose of sulfadoxine–pyrimethamine	3·00	Point estimate	Desai et al (2015); Kakuru et al (2016); and Kajubi et al (2019)^10^, ^11^, ^12^
Mean number of tablets per dose of dihydroartemisinin–piperaquine	9·00	Point estimate	Desai et al (2015); Kakuru et al (2016); and Kajubi et al (2019)^10^, ^11^, ^12^
**Costs from adverse health outcomes**			
Cost per inpatient day excluding medical supplies, US$ 2013 (to be adjusted for inflation; SD=35·88; Kenya; 95% CI)	37·12 (α=1·07, β=34·68; 1·10–131·20)	Gamma	Health-care facility costing, Kenya (n=4)
Cost per outpatient visit excluding medical supplies, US$ 2013 (to be adjusted for inflation; SD=1·28; Kenya; 95% CI)	3·73 (α=33·97, β=0·11; 2·60–5·10)	Gamma	Health-care facility costing, Kenya (n=4)
Adjustment factor for costs of inpatient and outpatient costs for Uganda	0·30	Point estimate	Ratio of Ministry of Health nurses' salaries, Uganda and Kenya
Incremental time in hospital for low birthweight *vs* normal birthweight in Kenya, days (95% CI; p value)	2·38 (1·76–3·00;p<0·0001)	Gamma	Kenya trial post-partum health utilisation form (low birthweight n=48, normal birthweight n=982)
**Parameters and assumptions to calculate cost per moderate anaemia and malaria in pregnancy episode**			
Short-term cost per low birthweight baby (Kenya), $ (95% CI)	121·86 (3·54–437·74)	NA	Probabilistic sensitivity analysis
Short-term cost per low birthweight baby (Uganda), $ (95% CI)	35·97 (1·04–129·19)	NA	Probabilistic sensitivity analysis
Short-term cost per low birthweight baby (meta-analysis), $ (95% CI)	78·91 (2·29–283·46)	NA	Probabilistic sensitivity analysis
Cost per moderate anaemia episode (Kenya), $ (95% CI)	10·01 (4·21–25·49)	NA	Probabilistic sensitivity analysis
Cost per moderate anaemia episode (Uganda), $ (95% CI)	4·78 (2·41–9·75)	NA	Probabilistic sensitivity analysis
Cost per moderate anaemia episode (meta-analysis), $ (95% CI)	7·39 (3·34–17·73)	NA	Probabilistic sensitivity analysis
Cost per malaria in pregnancy episode (Kenya), $ (95% CI)	12·40 (3·51–36·11)	NA	Probabilistic sensitivity analysis
Cost per malaria in pregnancy episode (Uganda), $ (95% CI)	5·49 (2·01–13·06)	NA	Probabilistic sensitivity analysis
Cost per malaria in pregnancy episode (meta-analysis), $ (95% CI)	8·95 (2·79–24·59)	NA	Probabilistic sensitivity analysis
**DALY calculations**			
Discount rate (range)	0·03 (0·00–0·05)	Point estimate	Assumption
Mean age, years (95% CI; Kenya)	23·40 (22·89–23·91)	Normal	Desai et al (2015)^12^
Mean age, years (95% CI; Uganda-I)	22·20 (21·33–23·07)	Normal	Kakuru et al (2016)^11^
Mean age, years (95% CI; Uganda-II)	23·00 (22·40–23·60)	Normal	Kajubi et al (2019)^10^
Life expectancy for women aged 20–24 years, years (Kenya, 2016)	53·30	Point estimate	Global health observatory data repository, WHO^25^
Life expectancy at birth, years (Kenya, 2016)	66·65	Point estimate	Global health observatory data repository, WHO^25^
Life expectancy for women aged 20–24 years, years (Uganda, 2016)	49·90	Point estimate	Global health observatory data repository, WHO^25^
Life expectancy at birth, years (Uganda, 2016)	62·50	Point estimate	Global health observatory data repository, WHO^25^
Length of disability: malaria during pregnancy, days (range)	3·5 (2·0–6·0)	Gamma	Assumption
Length of disability: malaria related anemia, days (range)	21·0 (14·0–42·0)	Gamma	Price et al (2001)^26^
Length of disability: low birthweight	Lifelong	NA	Global health observatory data repository, WHO^25^
Disability weight of severe infectious disease; acute episode (95% CI)	0·21 (0·14–0·30)	Log normal	GBD 2010 disability weights^17^*
Disability weight of mild maternal anaemia (95% CI)	0·004 (0·001–0·008)	Log normal	GBD 2015 disability weights^19^
Disability weight of moderate maternal anaemia (95% CI)	0·05 (0·03–0·08)	Log normal	GBD 2015 disability weights^19^
Disability weight of low birthweight	0·11 (0·07–0·14)	Log normal	GBD 2004 update, plus or minus 30%^18^
Case fatality rate from malaria during pregnancy, %†	0·33 (0·26–0·45)	Beta	Sicuri et al (2010)^21^
Case fatality rate from moderate or severe anaemia in pregnancy, %†	1·00 (0·80–1·20)	Beta	Brabin el al (2001), plus or minus 20%^20^
Case fatality rate from mild anaemia in pregnancy, %	0·00	Point estimate	Assumption

Mild anaemia was haemoglobin between 90 and <110 g/L, moderate anaemia was haemoglobin <90 g/L. Low birthweight was <2·5 kg. DALY=disability-adjusted life-year. IPTp-SP3=intermittent preventive treatment in pregnancy with three doses of sulfadoxine–pyrimethamine. IPTp-DP3=intermittent preventive treatment in pregnancy with three doses of dihydroartemisinin–piperaquine. IPTp-DP_monthly_=intermittent preventive treatment in pregnancy with monthly doses of dihydroartemisinin–piperaquine. IPTp-SP_monthly_=intermittent preventive treatment in pregnancy with monthly doses of sulfadoxine–pyrimethamine. α=shape parameter. β=rate parameter of the distribution. NA=not applicable. GBD=Global Burden of Disease study.

* GBD 2015 estimates for malaria were not used, but we used GBD 2012 estimate for severe episode of infectious disease.

† Distribution fitted by method of moments.

**Table 2:** Measures of effect

		**Kenya**	**Uganda-I**	**Uganda-II**	**Meta-analysis**	**Distribution**
**Child outcomes**						
Stillbirths						
	IPTp-SP3 group (α/β)	35·32 (16/437)	10·10 (1/98)	NA	30·80 (17/535)	Beta
	IPTp-DP3 group (α/β)	8·85 (4/448)	11·24 (1/88)	NA	9·24 (5/536)	Beta
	IPTp-SP_monthly_ group (α/β)	NA	NA	14·97 (5/329)	NA	Beta
	IPTp-DP_monthly_ group (α/β)	NA	10·20 (1/97)	5·90 (2/337)	NA	Beta
Neonatal deaths						
	IPTp-SP3 group (α/β)	27·46 (12/425)	0 (0/98)*	NA	22·43 (12/523)	Beta
	IPTp-DP3 group (α/β)	8·93 (4/444)	22·73 (2/86)	NA	11·19 (6/530)	Beta
	IPTp-SP_monthly_ group (α/β)	NA	NA	18·24 (6/323)	NA	Beta
	IPTp-DP_monthly_ group (α/β)	NA	30·93 (3/94)	11·87 (4/333)	NA	Beta
Low birthweight						
	IPTp-SP3 group (α/β)	32·18 (13/391)	132·65 (13/85)	NA	51·79 (26/476)	Beta
	IPTp-DP3 group (α/β)	53·14 (22/392)	127·91 (11/75)	NA	66·00 (33/467)	Beta
	IPTp-SP_monthly_ group (α/β)	NA	NA	89·78 (29/294)	NA	Beta
	IPTp-DP_monthly_ group (α/β)	NA	74·47 (7/87)	72·07 (24/309)	NA	Beta
**Maternal outcomes**						
Mild anaemia (Hb between 90 and <110 g/L) or moderate anaemia (Hb <90 g/L)						
	IPTp-SP3 group, mild/moderate (α_1_/α_2_/β)†	469·33/82·67 (176/31/168)	336·74/81·63 (33/8/57)	NA	441·86/82·45 (209/39/225)	Dirichlet
	IPTp-DP3 group, mild/moderate (α_1_/α_2_/β)†	502·70/56·76 (186/21/163)	311·11/11·11 (28/1/61)	NA	465·22/47·83 (214/22/224)	Dirichlet
	IPTp-SP_monthly_ group, mild/moderate (α_1_/α_2_/β)†	NA	NA	196·55 (171/699)‡	NA	Beta
	IPTp-DP_monthly_ group, mild/moderate (α_1_/α_2_/β)†	NA	282·83/50·51 (28/5/66)	98·45 (89/815)‡	NA	Dirichlet or beta
Clinical malaria						
	IPTp-SP3 group (α_1_/α_2_/α_3_/β)§	123·46 (52/4/0/430)	386·79 (23/9/0/74)	NA	170·61 (75/13/0/504)	Dirichlet
	IPTp-DP3 group (α_1_/α_2_/α_3_/β)§	26·80 (8/1/1/475)	127·66 (10/1/0/83)	NA	43·17 (18/2/1/558)	Dirichlet
	IPTp-SP_monthly_ group (α_1_/α_2_/α_3_/β)§	NA	NA	221·90 (75/263)¶	NA	Beta
	IPTp-DP_monthly_ group (α_1_/α_2_/α_3_/β)§	NA	0 (0/0/0/99)	8·60 (3/346)¶	NA	Dirichlet or beta

Data are shown as events per 1000 women following the decision tree, as well as α (events) and β (no events). Low birthweight was <2·5 kg. IPTp-SP3=intermittent preventive treatment in pregnancy with three doses of sulfadoxine–pyrimethamine. NA=not applicable. IPTp-DP3=intermittent preventive treatment in pregnancy with three doses of dihydroartemisinin–piperaquine. IPTp-SP_monthly_=intermittent preventive treatment in pregnancy with monthly doses of sulfadoxine–pyrimethamine. IPTp-DP_monthly_=intermittent preventive treatment in pregnancy with monthly doses of dihydroartemisinin–piperaquine. Hb=haemoglobin.

* Transformation for probabilistic sensitivity analysis: α_t_=α + 0·1, β_t_=β – 0·1.

† α_1_=mild anaemia events; α_2_=moderate anaemia events; β=no events.

‡ Only α_2_=moderate anaemia and β=no events are given for Uganda-II.

§ α_1_=one episode of clinical malaria; α_2_=two episodes of clinical malaria; α_3_=three episodes of clinical malaria; β=no events.

¶ Only α=episodes of malaria and β=no events are given for Uganda-II.

Analysis of the observations of nurses' time to administer the intervention and the meta-analysis of outcomes was done using STATA version 15, and the international procurement data analysis, health-care facility costing studies, and the cost-effectiveness modelling were done in Microsoft Excel (using Visual Basic for Applications for the probabilistic sensitivity analysis). Our analytical and reporting methodology was guided by the Gates Reference Case.^22^

### Role of the funding source

The funder of the study had no role in study design, data collection, data analysis, data interpretation, or writing of the report. SF and KH had full access to all the data in the study and had final responsibility for the decision to submit for publication.

## Results

The meta-analysis results are shown in the appendix (pp 9–12). Maternal malaria and moderate anaemia outcomes were better with IPTp-DP3 than with IPTp-SP3, but there was no difference in mild anaemia. For the child health outcomes, stillbirth and neonatal death were better with IPTp-DP3, but low birthweight was marginally better in the IPTp-SP3 group. Here we report deterministic results, followed by the CrIs from the probabilistic sensitivity analysis.

The incremental effectiveness per 1000 pregnant women of IPTp-DP3 versus IPTp-SP3 was 892 DALYs averted (95% CrI 274 to 1517; table 3). The incremental effectiveness of IPTp-DP_monthly_ compared with IPTp-DP3 was –44 DALYS averted (−1575 to 1472) and compared with IPTp-SP3 was –646 DALYs averted (−1976 to 528). The negative DALYs can be attributed to a small, non-significant increase in child mortality outcomes in both IPTp-DP groups in the Uganda-I trial, which was a small trial (n=300) with insufficient statistical power to detect a difference in mortality outcomes. Finally, the incremental effectiveness of IPTp-DP_monthly_ compared with IPTp-SP_monthly_ was 534 DALYs averted (−141 to 1233). The health outcomes contributing most to a positive difference in DALYs averted differed among the three trials. Stillbirth and neonatal death were the main drivers for DALYs averted with IPTp-DP3 versus IPTp-SP3 (pooled data) and for IPTp-DP_monthly_ versus IPTp-SP_monthly_ (Uganda-II), while low birthweight was most relevant for the Uganda-I trial, comparing IPTp-DP_monthly_ with IPTp-DP3 or IPTp-SP3.

**Table 3:** Cost, outcome, and cost-effectiveness results

	**Kenya (IPTp-DP3 *vs* IPTp-SP3)**	**Uganda-I (IPTp-DP3 *vs* IPTp-SP3)**	**Uganda-I (IPTp-DP**_monthly_ vs **IPTp-SP3)**	**Uganda-I (IPTp-DP**_monthly_ vs **IPTp-DP3)**	**Uganda-II (IPTp-DP**_monthly_ vs **IPTp-SP**_monthly_**)**	**Meta-analysis (IPTp-DP3 *vs* IPTp-SP3)**
**Costs**						
Incremental costs per 1000 women including costs from consequences, US$ 2018	8201 (8230 [3176 to 16 688])	5467 (5403 [−1918 to 11 623])	12 406 (12 391[508 to 22 789])	6939 (6988 [−1934 to 12 989])	13 427 (13 310 [4994 to 22 895])	7051 (7080 [2653 to 13 038])
**Outcomes averted**						
Low birthweight cases averted per 1000 women	−22 (−22 [−50 to 4])	8 (8 [−89 to 100])	60 (60 [−22 to 144])	52 (52 [−30 to 140])	18 (18 [−24 to 60])	−16 (−16 [−44 to 13])
Stillbirth cases averted per 1000 women	26 (26 [8 to 47])	−1 (−1 [−33 to 31])	0 (0 [−30 to 31])	1 (1 [−30 to 33])	9 (9 [−5 to 25])	22 (22 [6 to 39])
Neonatal death averted per 1000 women	18 (18 [2 to 36])	−22 (−21 [−62 to −1])	−31 (−29 [−71 to −5])	−8 (−8 [−56 to 39])	6 (6 [−12 to 26])	11 (11 [−4 to 26])
Mild anaemia cases averted per 1000 women	−33 (−34 [−105 to 39])	26 (26 [−104 to 157])	54 (55 [−73 to 182])	28 (28 [−101 to 160])	N/A	−23 (−24 [−89 to 41])
Moderate anaemia cases averted per 1000 women	26 (26 [−10 to 63])	71 (71 [19 to 137])	31 (31 [−37 to 103])	−39 (−39 [−92 to 5])	98 (98 [64 to 130])	35 (35 [3 to 66])
Clinical malaria cases averted per 1000 women	97 (97 [64 to 130])	259 (259 [145 to 373])	386 (386 [296 to 481])	127 (127 [69 to 201])	213 (213 [169 to 259])	127 (128 [93 to 162])
**DALYs averted**						
Total DALYs averted per 1000 women	1220 (1183 [498 to 1926])	−602 (−557 [−1913 to 553])	−646 (−602 [−1976 to 528])	−44 (−44 [−1575 to 1472])	534 (522 [−141 to 1233])	892 (873 [274 to 1517])
**ICER**						
Total cost per DALY averted including costs from consequences, US$ 2018	7 (7 [2 to 22])	−9 (−10 [−110 to 93])	−19 (−21 [−219 to 192])	−158 (−159 [−146 to 166])*	25 (26 [−151 to 224])	8 (8 [2 to 29])
Likelihood of being cost-effective (shown as percentage of 10 000 simulations that were cost-effective)						
Simulations cost-effective at CET1† (Kenya $79·8, Uganda $30·6), %	99·88%	10·15%	4·82%	35·17%	58·75%	99·43%/97·64%§
Simulations cost-effective at CET2† (Kenya $676·5, Uganda $411·8), %	99·96%	15·17%	13·39%	46·36%	92·66%	99·74%/99·73%§
Simulations cost-effective at CET3† (Kenya $1273·1, Uganda $793·0), %	99·96%	15·45%	13·88%	46·86%	93·24%	99·74%/99·74%§
Simulations cost-effective at CET4‡ (Kenya $520·2, Uganda $124·0), %	99·96%	13·87%	10·97%	44·19%	89·20%	99·73%/99·51%§
Simulations cost-effective at CET5‡ (Kenya $685·5, Uganda $163·2), %	99·96%	14·24%	11·66%	44·92%	90·34%	99·74%/99·64%§

Results are presented as base case result (mean probabilistic sensitivity analysis result [95% credibility estimates]), unless otherwise stated. All numbers, with the exception of percentages, are presented without decimals to ease interpretation of the results. The base case result represents the results of the deterministic base case analysis, where for each parameter the best estimate was used. The numbers in parentheses stem from the probabilistic sensitivity analysis, which was done using 10 000 simulations. DALYs and ICERs were estimated including clinical malaria, anaemia, stillbirth, low birthweight, and neonatal death. Mild anaemia was haemoglobin between 90 and <110 g/L, moderate anaemia was haemoglobin <90 g/L. Low birthweight was <2·5 kg. A negative effect size number (negative difference in number of events of a certain outcome or DALYs averted) indicates the intervention led to more events or DALYs—ie, the intervention was less effective than the comparison regimen for that particular outcome, leading to a negative incremental cost-effectiveness ratio. IPTp-DP3=intermittent preventive treatment in pregnancy with three doses of dihydroartemisinin–piperaquine. IPTp-SP3=intermittent preventive treatment in pregnancy with three doses of sulfadoxine–pyrimethamine. IPTp-DP_monthly_=intermittent preventive treatment in pregnancy with monthly doses of dihydroartemisinin–piperaquine. IPTp-SP_monthly_=intermittent preventive treatment in pregnancy with monthly doses of sulfadoxine–pyrimethamine. N/A=not available. DALY=disability-adjusted life-year. ICER=Incremental cost-effectiveness ratio. CET=cost-effectiveness threshold.

* The median value for the ICER was −2·93, which is included in the credibility interval.

† Country-level CETs from Woods et al (2016).^27^

‡ Country-level CETs from Ochalek et al (2018).^28^

§ CET Kenya/CET Uganda.

The incremental cost per 1000 pregnant women of IPTp-DP3 compared with IPTp-SP3 was $7051 (95% CrI 2653 to 13 038). The incremental cost of IPTp-DP_monthly_ compared with IPTp-DP3 was $6939 (−1934 to 12 989) and compared with IPTp-SP3 was $12 406 (508 to 22 789). Finally, the incremental cost of IPTp-DP_monthly_ compared with IPTp-SP_monthly_ was $13 427 (4994 to 22 895).

The ICER of IPTp-DP3 compared with IPTp-SP3 was $8 (95% CrI 2 to 29) per DALY averted. IPTp-DP_monthly_ was both more costly and less effective than IPTp-DP3 and IPTp-SP3 if all outcomes are included. The ICER of IPTp-DP_monthly_ compared with IPTp-SP_monthly_ was $25 (−151 to 224) per DALY averted.

**Figure 1:**
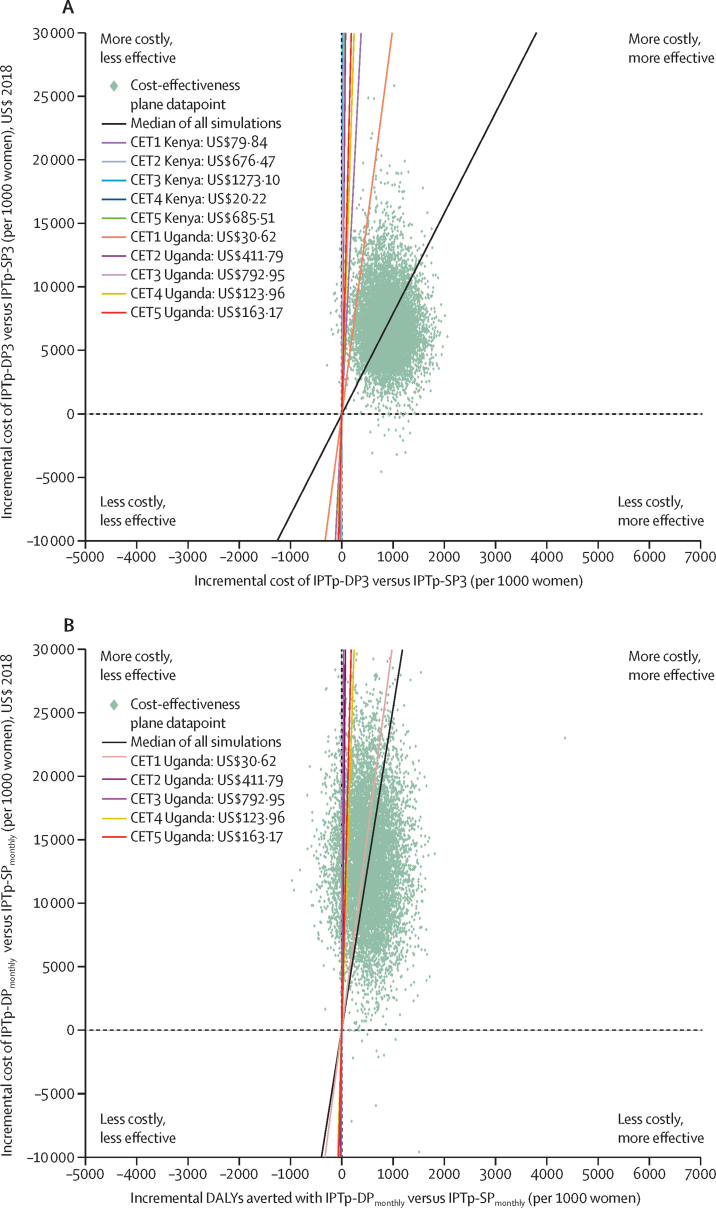
Cost-effectiveness planes for IPTp-DP3 versus IPTp-SP3 and IPTp-DP_monthly_ versus IPTp-SP_monthly_ Cost-effectiveness planes in a hypothetical cohort of 1000 pregnant women for IPTp-DP3 versus IPTp-SP3, using meta-analysis pooled data (A), and IPTp-DP_monthly_ versus IPTp-SP_monthly_, using data from the Uganda-II trial (B). Further cost-effectiveness planes comparing IPTp-DP3 with IPTp-SP3, IPTp-DP_monthly_ with IPTp-DP3, IPTp-DP_monthly_ with IPTp-SP3 (Uganda-I), and IPTp-DP3 with IPTp-SP3 (Kenya) are given in the appendix (pp 13–17). The cost-effectiveness planes display the results of the probabilistic sensitivity analysis using 10 000 simulations. Each data point represents one simulation, where the value for each parameter is sampled from a distribution. The CETs were estimated using country-specific CETs published by Woods et al (2016)^27^ and Ochalek et al (2018),^28^ adjusted for inflation. IPTp-DP3=intermittent preventive treatment in pregnancy with three doses of dihydroartemisinin–piperaquine. IPTp-SP3=intermittent preventive treatment in pregnancy with three doses of sulfadoxine–pyrimethamine. IPTp-DP_monthly_=intermittent preventive treatment in pregnancy with monthly doses of dihydroartemisinin–piperaquine. IPTp-SP_monthly_=intermittent preventive treatment in pregnancy with monthly doses of sulfadoxine–pyrimethamine. CET=cost-effectiveness threshold. DALY=disability-adjusted life-year.

Using 10 000 simulations for the probabilistic sensitivity analysis, the simulation points were plotted on the cost-effectiveness planes and the likelihood of a scenario being cost-effective was calculated for each cost-effectiveness threshold. Comparing IPTp-DP3 with IPTp-SP3, the percentage of simulations indicating a cost-effective result was more than 97·6% for all cost-effectiveness thresholds. In contrast, only 35·2–46·9% of simulations comparing IPTp-DP_monthly_ with IPTp-DP3 and 4·8–13·9% of simulations comparing IPTp-DP_monthly_ with IPTp-SP3 were judged cost-effective against the cost-effectiveness thresholds. For the five cost-effectiveness thresholds considered, 58·8–93·2% of simulations comparing IPTp-DP_monthly_ with IPTp-SP_monthly_were found to be cost-effective (figure 1, table 3, appendix pp 13–17).

**Figure 2:**
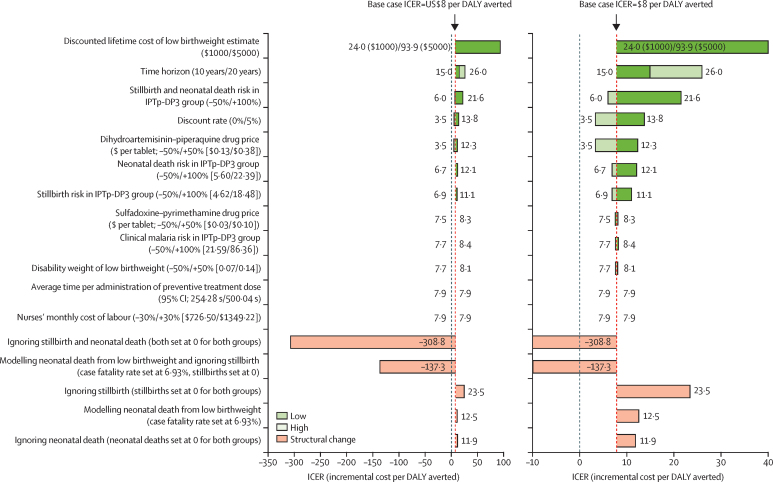
Deterministic sensitivity analysis; tornado diagram of IPTp-DP3 versus IPTp-SP3 using pooled data The right side of the figure shows a magnified version of the graph for visual clarity. The base case ICER (US$8 per DALY averted) is shown with black arrows in the graph. 12 single parameters were varied individually using lower and higher estimates, shown with green bars on the graph, and there were five more structural changes to the model, reflecting that the outcomes driving the cost-effectiveness vary among the three trials (Kenya, Uganda-I, and Uganda-II); these changes are shown with orange bars. IPTp-DP3=intermittent preventive treatment in pregnancy with three doses of dihydroartemisinin–piperaquine. IPTp-SP3=intermittent preventive treatment in pregnancy with three doses of sulfadoxine–pyrimethamine. ICER=incremental cost-effectiveness ratio. DALY=disability-adjusted life-year.

In the deterministic sensitivity analysis, for IPTp-DP3 versus IPTp-SP3 (pooled data, base case ICER $8 per DALY averted) all but three variations produced ICERs that remained cost-effective relative at all cost-effectiveness thresholds (figure 2). Our base case analysis only included the short-term costs of low birthweight; therefore, we modelled the effect of including an arbitrary cost of low birthweight on the ICER in the pooled analysis. Using an incremental discounted lifetime cost per low birthweight baby of $1000 changed the ICER of IPTp-DP3 compared with IPTp-SP3 to $24 per DALY averted and a cost of $5000 changed the ICER to $94 per DALY averted; IPTp-DP3 remained cost-effective relative to all cost-effectiveness thresholds when using a cost of $1000 per low birthweight baby, and relative to all but the lowest cost-effectiveness threshold from each country when using a cost of $5000. Omitting stillbirth and neonatal death from the outcome measurement and then modelling neonatal death from low birthweight (with a case fatality rate of 6·93%)^29^ resulted in IPTp-DP3 being less effective and more costly than IPTp-SP3. Conversely, when comparing IPTp-DP_monthly_ with IPTp-DP3 (appendix p 18), for which the base case found IPTp-DP_monthly_ to be less effective and more costly than IPTp-DP3 (ICER –$158 per DALY averted), the sensitivity analysis scenario produced an ICER of $26 per DALY averted, when investigating the same changes. Thus, halving the risk of neonatal death, stillbirth, or both in the IPTp-DP_monthly_ group, omitting neonatal death, and modelling neonatal death from low birthweight, all led to IPTp-DP_monthly_ being more cost-effective than IPTp-DP3 for all or most cost-effectiveness thresholds. Finally, for IPTp-DP_monthly_ versus IPTp-SP_monthly_, although some deterministic changes resulted in higher ICERs, IPTp-DP_monthly_ remained cost-effective for at least two cost-effectiveness thresholds with all changes (appendix p 19). The largest changes to the ICER arose from doubling the risk of both stillbirth and neonatal death in the IPTp-DP_monthly_ group (ICER $411 per DALY averted), followed by omitting both stillbirth and neonatal death in the model ($136 per DALY averted), and by omitting stillbirth and neonatal death plus modelling neonatal death from low birthweight ($101 per DALY averted).

## Discussion

This study brings together all of the currently available evidence on the cost-effectiveness of IPTp-DP in preventing malaria in pregnancy and its consequences for the health of the mother and her baby. Although at first glance our findings paint a slightly mixed picture of the cost-effectiveness of IPTp-DP, looking at the base case analysis together with sensitivity analyses and understanding of trial contexts provides a convincing interpretation. IPTp-DP3 was highly cost-effective compared with IPTp-SP3, with a low estimated ICER of $8 per DALY averted and a likelihood of being cost-effective of at least 97·6%, with respect to all cost-effectiveness thresholds. When excluding both neonatal deaths and stillbirths in the sensitivity analysis, the difference in DALYs between IPTp-DP3 and IPTp-SP3 became negative, as did the ICER (lower efficacy at a higher cost), which is primarily caused by a slightly higher number of cases of low birthweight in the IPTp-DP3 group when compared to IPTp-SP3. By contrast, IPTp-DP_monthly_ was less effective and at higher cost than IPTp-DP3 in the Uganda-I trial, producing a negative ICER, mainly due to higher numbers of neonatal deaths in the IPTp-DP_monthly_ group, but the sensitivity scenarios excluding or modelling neonatal death generated highly cost-effective ICERs ranging from $24 to $43 per DALY averted. Lastly, IPTp-DP_monthly_ versus IPTp-SP_monthly_ produced a highly cost-effective ICER of $25 per DALY averted. For this comparison, the likelihood of cost-effectiveness in the probabilistic sensitivity analysis was 58·6% for the lowest cost-effectiveness threshold ($30·6) and ranged from 89·2% to 93·2% for the second to fifth thresholds. The cost-effectiveness remained robust in the deterministic sensitivity analysis, with no changes producing a negative ICER, and some changes producing higher but still cost-effective ICERs.

Over time, national policies have moved from IPTp-SP3 to IPTp-SP_monthly_, enabling the Uganda-I trial to incorporate an IPTp-DP_monthly_ group and the most recent trial, Uganda-II, to study IPTp-DP_monthly_ versus IPTp-SP_monthly_. We opted to present the cost-effectiveness of the three trials and their pooled estimates together to provide decision makers with a comprehensive overview of the current evidence on the cost-effectiveness of IPTp-DP. However, the difference in the sizes of the trials, and the implications for the interpretation of the results create some challenges and caveats.

In principle, all relevant outcomes should be included in a cost-effectiveness analysis (ie, the same criteria should be applied consistently when selecting the outcomes to include). In this case, the primary outcome (DALYs averted by the intervention) is a composite outcome, generated by adding together multiple health outcomes (eg, low birthweight, maternal anaemia, and malaria morbidity and mortality) measured in pregnant women and their babies. While in all three trials IPTp-DP was found to be highly efficacious, this efficacy varied for different outcomes across the three trials, with IPTp-DP leading to fewer neonatal deaths and stillbirths in the Kenya and Uganda-II trials, and fewer low birthweight babies in the Uganda-I and Uganda-II trials. These differences were particularly relevant when comparing IPTp-DP_monthly_ with IPTp-SP3, because the sample size in the Uganda-I trial was much smaller (n=100 per group), introducing more uncertainty than for the other comparisons. In the Kenya trial (n=500 per group), the pooled data, and the Uganda-II trial, mortality outcomes (neonatal death and stillbirth) were the main drivers of the cost-effectiveness of IPTp-DP. However, in the smaller Uganda-I trial there were slightly more neonatal deaths in the IPTp-DP_monthly_ group (three deaths *vs* two in the IPTp-DP3 group and none in the IPTp-SP3 group), which becomes magnified when cost-effectiveness is modelled in a hypothetical cohort of 1000 women. When these deaths are included in our model and DALY calculation, the IPTp-DP groups cannot be effective in the Uganda-I trial, and therefore not cost-effective, despite a beneficial effect in the IPTp-DP groups for low birthweight, clinical malaria, and mild maternal anaemia. Modelling neonatal mortality from low birthweight instead, provides some insight into the potential ICER. An additional death at birth contributes so much more to the calculation of total DALYs that it offsets all other outcomes. One death at birth in the Uganda-I trial contributes 27·6 DALYs compared with only 2·9 DALYs per low birthweight case and less than 0·3 DALYs for all other outcomes. For the aggregated data model, we were faced with the issue that when excluding mortality outcomes, there were more cases of low birthweight (15·6 more cases per 1000 pregnant women) in the IPTp-DP3 group than in the IPTp-SP3 group. Desai and colleagues suggested that the improved survival in the IPTp-DP3 group might have resulted in more livebirths with lower birthweights.^12^

The deterministic sensitivity analysis in particular highlights the importance of the risk of stillbirth and neonatal death for the cost-effectiveness of IPTp-DP. Although there is some heterogeneity in the cost-effectiveness, the analyses based on the pooled data and the Uganda-II trial data both produce a highly cost-effective ICER of $8 or $25 per DALY averted. Their cost-effectiveness planes reveal a high proportion of simulations being cost-effective for the different cost-effectiveness thresholds and the ICERs are robust to different assumptions being tested in the deterministic sensitivity analysis. When we model the cost-effectiveness of IPTp-DP_monthly_ compared with IPTp-DP3 (Uganda-I) by applying a case fatality rate of 6·93% to the low birthweight cases, we estimate a highly cost-effective ICER of $24 per DALY averted. Our findings, therefore, suggest that both IPTp-DP regimens are likely to be highly cost-effective in settings of comparable endemicity and sulfadoxine–pyrimethamine resistance. Nevertheless, to be able to confidently judge the cost-effectiveness of IPTp-DP and decide on the correct regimen, larger trials with the monthly regimens in settings with similar or higher levels of sulfadoxine–pyrimethamine resistance are needed.

Our study had several other limitations. Primary cost data collection to calculate cost savings of the intervention was not undertaken in Uganda, other than ex-post collection of nurses' salary costs. Instead, we have adjusted the Kenyan costs to Ugandan price levels using a ratio of the average nurses' salary in the two countries. The drug costs of both sulfadoxine–pyrimethamine and dihydroartemisinin–piperaquine were calculated from international procurement databases and are not expected to vary between the two countries because of international procurement for malaria control programmes. Although this calculation probably represents the true cost of the widely used sulfadoxine–pyrimethamine, it might not reflect the actual cost that would be paid if IPTp-DP was to be widely adopted as national policy, as dihydroartemisinin–piperaquine is currently procured in much smaller quantities. Changing to IPTp-DP in several countries would probably reduce the costs of dihydroartemisinin–piperaquine, rendering both IPTp-DP3 and IPTp-DP_monthly_ more cost-effective than estimated in this study. Long-term costs of low birthweight babies in low-income and middle-income countries are generally unknown; therefore we only included the incremental short-term cost of hospitalisation of low birthweight compared with normal birthweight babies measured in the trial population. This costing greatly underestimates the lifetime costs of low birthweight, which is particularly relevant for the pooled data, where the IPTp-SP group had fewer low birthweight cases. We addressed this possible underestimation in the sensitivity analysis by exploring the changes to the ICER when including two very high arbitrary values of $1000 and $5000 as the discounted incremental lifetime cost of low birthweight. In both cases, the ICERs remained relatively robust and highly cost-effective at $24 and $93 per DALY averted.

In the Kenya trial, approximately 10% of the babies were not weighed, and Desai and colleagues^12^ did not report whether there were any differences between weighed and unweighed babies, nor did the cost-effectiveness analysis explore a potential underestimation or overestimation in the number of low birthweight babies in a sensitivity analysis.

We have described the rationale for using the same analytical metrics for all trials. Although a strength of this study is that it presents the full available evidence to decision makers, the execution and interpretation of the analysis require a careful reflection on the differences in study design and the results of the individual trials to interpret the cost-effectiveness results and sensitivity analysis.

In summary, our data suggest that IPTp-DP3 and IPTp-DP_monthly_ are likely to be highly cost-effective in areas of high malaria transmission and sulfadoxine–pyrimethamine resistance, in HIV-negative pregnant women with a high uptake of long-lasting insecticidal nets. Nevertheless, before a policy change is advocated, we recommend further research into the efficacy of IPTp-DP_monthly_ in preventing malaria infection during pregnancy and its sequelae. Ideally, further research should also include malaria prevention in HIV-positive women, who continue to rely solely on co-trimoxazole.
