# Supplementary material for: Cost-effectiveness of intermittent preventive treatment with dihydroartemisinin–piperaquine for malaria during pregnancy: an analysis using efficacy results from Uganda and Kenya, and pooled data
Source: Lancet Glob Health. 2020 Oct 30;8(12):e1512–23. doi: 10.1016/S2214-109X(20)30369-7 (PMC7686013; doi:10.1016/S2214-109X(20)30369-7)
Supplement: Supplementary appendix [file mmc1.pdf]

THE LANCET
Global Health

Supplementary appendix

This appendix formed part of the original submission and has been peer reviewed. We post it as supplied by the authors.

Supplement to: Silke Fernandes S, Were V, Gutman J, et al. Cost-effectiveness of intermittent preventive treatment with dihydroartemisinin–piperaquine for malaria during pregnancy: an analysis using efficacy results from Uganda and Kenya, and pooled data. *Lancet Glob Health* 2020; published online Oct 30. http://dx.doi.org/10.1016/S2214-109X(20)30369-7.

Is intermittent preventive treatment during pregnancy with dihydroartemisinin-piperaquine cost-effective? Current evidence using efficacy results from Uganda, Kenya and pooled data

Silke Fernandes, MSc [1]; Vincent Were, MSc[2]; Julie Gutman, MD, MSc[6]; Professor Grant Dorsey, PhD[5]; Abel Kakuru, MD[3]; Meghna Desai, PhD, MPH[6]; Simon Kariuki, PhD[2]; Professor Moses R. Kamya, PhD[7]; Professor Feiko O. ter Kuile, PhD[4]; Professor Kara Hanson, ScD[1]

1 London School of Hygiene and Tropical Medicine, London, United Kingdom
2 Kenya Medical Research Institute, Centre for Global Health Research, Kisumu, Kenya
3 Infectious Diseases Research Collaboration, Kampala, Uganda
4 Liverpool School of Tropical Medicine, Liverpool, United Kingdom
5 Department of Medicine, University of California, San Francisco, USA
6 Malaria Branch, Division of Parasitic Diseases and Malaria, Center for Global Health, Centers for Disease Control and Prevention, Atlanta, GA, USA
7 School of Medicine, Makerere University College of Health Sciences, Kampala, Uganda

Supplementary appendix

Table of content:

Appendix 1: Comparison arms of the three trials 2

Appendix 2: Overview of trial sites, trial context and trial designs 3

Appendix 3: Trial baseline characteristics and key outcomes 4

Appendix 4: Decision tree of child outcomes including stillbirth, neonatal death and low birth weight 5

Appendix 5: Decision tree of maternal anaemia 7

Appendix 6: Decision tree of maternal clinical malaria 8

Appendix 7: Meta-analysis results child outcomes 9

Appendix 8: Meta-analysis results maternal outcomes 11

Appendix 9: Cost effectiveness planes Uganda-I data, IPTp-DP3 versus IPTp-SP3 13

Appendix 10: Cost effectiveness planes Uganda-I data, IPTp-$DP_{monthly}$ versus IPTp-DP3 14

Appendix 11: Cost effectiveness planes Uganda-I data, IPTp-$DP_{monthly}$ versus IPTp-SP3 15

Appendix 12: Cost effectiveness planes Kenya data, IPTp-DP3 versus IPTp-SP3 16

Appendix 13: Deterministic sensitivity analysis: Tornado diagram of IPTp-$DP_{monthly}$ versus DP3 using Uganda-I data 18

Appendix 14: Deterministic sensitivity analysis: Tornado diagram of IPTp-$DP_{monthly}$ versus $SP_{monthly}$ using Uganda-II data 19

Appendix 1: Comparison arms of the three trials

	IPTp-DP Intervention(s)	IPTp-SP Control
Kenya, Desai *et al.*, 2015[1]	DP3	SP3
Uganda-I, Kakuru *et al.*, 2016[2]	DP3 & $DP_{monthly}$	SP3
Uganda-II, Kajubi *et al.*, 2019[3]	$DP_{monthly}$	$SP_{monthly}$

Abbreviations: IPTp=Intermittent preventive treatment during pregnancy; SP3 or $SP_{monthly}$=three or monthly doses of Sulfadoxine-Pyrimethamine; DP3 or $DP_{monthly}$=three or monthly doses of Dihydroartemisinin-Piperaquine

Appendix 2: Overview of trial sites, trial context and trial designs

Author	Desai et al., 2015	Kakuru et al., 2016	Kajubi et al., 2019
Location	Siaya, Western Kenya	Tororo, Eastern Uganda	Busia district, Eastern Uganda
Population	mostly rural<br>833,760 (Siaya county)	mostly rural<br>517,082 (Tororo district), Census 2014	mostly rural<br>323,662 (Busia district), Census 2014
Malaria transmission	Lake epidemic, moderate to high year-round transmission<br>high SP resistance	Lake epidemic, high transmission<br>high SP resistance	Lake epidemic, high transmission<br>high SP resistance
% pregnant women sleeping under ITN last night	Lake endemic (MIS, 2015):<br>77.6%	Bukedi region (MIS 2018/2019):<br>84.1%	Bukedi region (MIS 2018/2019):<br>84.1%
IPTp-SP coverage	Lake endemic (MIS, 2015):<br>2 or more doses: 54.7%<br>3 or more doses: 35.3%	Bukedi region (MIS 2018/2019):<br>2 or more doses: 77.4%<br>3 or more doses: 40.2%	Bukedi region (MIS 2018/2019):<br>2 or more doses: 77.4%<br>3 or more doses: 40.2%
Trial name in paper	Kenya	Uganda-I	Uganda-II
Arms	IPTp-SP3, IPTp-DP3 *	IPTp-SP3, IPTp-DP3 and IPTp-Dpmonthly	IPTp-SPmonthly and IPTp-Dpmonthly
Sample size	N=1031	N=300	N=782
Primary outcome(s)	Malaria infection at delivery, a composite measure of peripheral or placental parasitaemia detected by placental histology, microscopy or rapid diagnostic tests	Histopathologically confirmed placental malaria	prevalence of composite measure of adverse birth outcomes comprised of low birth weight, preterm birth, small for gestational age
Secondary outcome(s)	- incidence of malaria infection<br>- incidence clinical malaria during pregnancy<br>- prevalence of adverse newborn morbidity at birth (composite measure preterm birth, low birth weight, small for gestational age)<br>- anaemia (<100g/l) during pregnancy and at delivery	-Incidence of symptomatic malaria<br>- prevalence of parasitemia<br>- prevalence of anemia (<110g/l)<br>- prevalence parasitaemia at delivery<br>- adverse birth outcomes including stillbirth, low birth weight, preterm birth, congenital anomaly and a composite of these	- incidence of symptomatic malaria<br>- prevalence of parasitaemia<br>- prevalence of anaemia (<100g/l)<br>- prevalence of placental malaria<br>- individual measures of adverse birth outcomes listed above plus stillbirth, spontaneous abortion, neonatal death and composite of fetal and neonatal death
Sample size calculation	To detect a 50% decrease in malaria infection at delivery (12% to 6%) with 80% power at and α of 0.025; 20% loss to follow up. 12% prevalence was conservative, estimated to be more close to 18%	80% power to show a 33% lower prevalence of placental malaria at an α of 0.025 in the IPTp-DP arms assuming a 62% baseline prevalence of placental malaria (based on previous data) in the IPTp-SP group	80% power to show a 30% lower relative risk in the primary outcome at an α of 0.025. Loss to follow-up assumed at 5%.

* a third arm of intermittent screening followed by treatment with DP was omitted from this analysis

Appendix 3: Trial baseline characteristics and key outcomes

Author	Desai et al., 2015		Kakuru et al., 2016			Kajubi et al., 2019	
Location	Siaya, Western Kenya		Tororo, Eastern Uganda			Busia district, Eastern Uganda	
Trial name in paper	Kenya		Uganda-I			Uganda-II	
Arms	IPTp-SP3 and IPTp-DP3 *		IPTp-SP3, IPTp-DP3 and IPTp-Dpmonthly			IPTp-SPmonthly and IPTp-Dpmonthly	
Sample size	N=1031		N=300			N=782	
**Baseline characteristics**							
	IPTp-SP3	IPTp-DP3	IPTp-SP3	IPTp-DP3	IPTp-DPmonthly	IPTp-SPmonthly	IPTp-DPmonthly
Age at enrolment (years)	23.5	23.4	21.3	22.2	22.6	23	23
Gestational age (weeks)	22.8	23	15.2	15.4	15.5	15.4	15.0
Gravidity (% primi and secundigravidae)	57%	51%	69.8%	64.9%	64.0%	47.8%	50.6%
Weight (kg)	61.5	61.8	55.4	55.6	55.5	Not provided	Not provided
Height (cm)	164.3	164.3	162.8	162.5	162.3	Not provided	Not provided
Hemoglobin (g/l)	105	106	118	119	120	115	114
Malaria infection by PCR (%)	33%	31%					
Detection of malaria parasites by LAMP (%)			55.7%	59.1%	57.0%	50.4%	52.2%
**Key outcomes summary**							
Maternal anaemia (haemoglobin <110g/l)	176/375 (47%)	186/370 (50%)	33/98 (33.7%)	28/90 (31%)	28/99 (28.3%)	171/870 (20%)[#]	89/904 (10%)[#]
Symptomatic malaria no. of events (incidence per person-year at risk)	57 (0.38)	9 (0.06)	41 (0.95)	12 (0.31)	0	75 (0·52)	3 (0·02)
Stillbirths	16/453 (4%)	4/452 (1%)	1/99 (1.0%)	1/89 (1.1%)	1/98 (1.0%)	5/334 (2%)	2/339 (1%)
Low birth weight	18/409 (4%)	22/414 (5%)	14/99 (14.1%)	14/89 (15.7%)	8/98 (8.2%)	29/329 (9%)	24/337 (7%)
Preterm delivery	21/451 (5%)	23/448 (5%)	8/99(8.1%)	11/89 (12.4%)	5/98 (5.1%)	24/329 (7%)	16/337 (5%)
Neonatal death	12/453 (3%)	4/452 (1%)	0/98	2/88 (2.3%)	3/97 (3.1%)	6/329 (2%)	4/337 (1%)

* a third arm of intermittent screening follwed by treatment with DP was omitted from this analysis

[#] hemoglobin <100g/l

Appendix 4: Decision tree of child outcomes including stillbirth, neonatal death and low birth weight

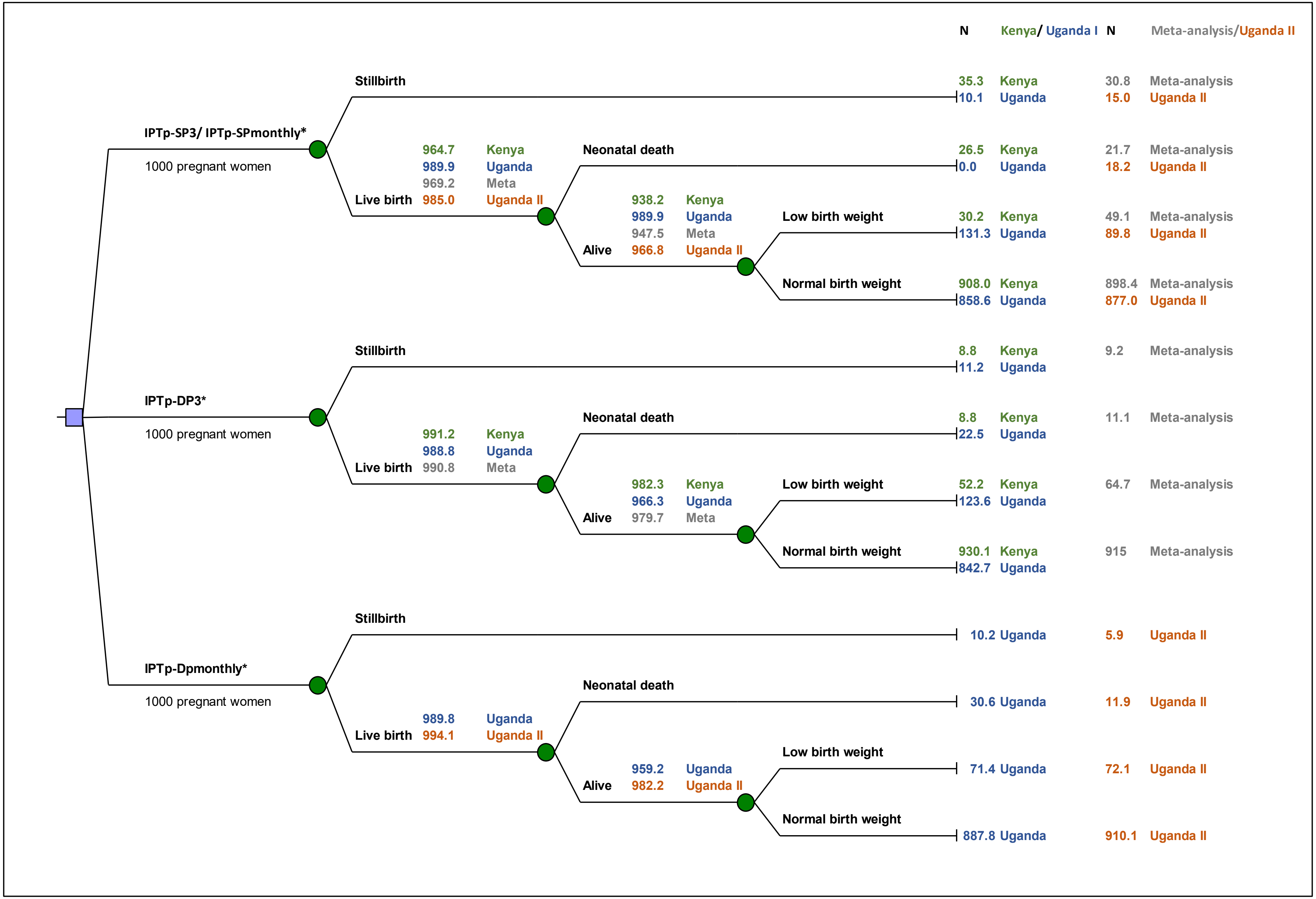

Appendix 4: Decision tree for child outcomes showing up to three arms branching into stillbirth, neonatal death and low birth weight. Neonatal death was given priority over low birth weight, as in Kenya not all babies who died during the first month had their birthweight recorded. The numbers as we move through the decision tree are shown per 1000 pregnant women per arm for Kenya (in green), Uganda (in blue), the meta-analysis (in grey) and Uganda-II (in red). The decision trees for maternal outcomes including clinical malaria and anaemia can be found in the supplementary appendix.

*Not all arms were included in all countries. For details see Appendix 1. The comparison arms were i) for Kenya: IPTp-DP3 vs IPTp-SP3[1]; ii) for Uganda-I: IPTp-$DP_{monthly}$, IPTp-DP3 vs IPTp-SP3 [2]; iii) for Uganda-II: IPTp-$DP_{monthly}$ vs IPT-$SP_{monthly}$ [3] and iv) for meta: IPTp-DP3 vs IPTp-SP3 [1, 2]

Abbreviations: DP3=3 doses of Dihydroartemisinin-piperaquine; $DP_{monthly}$= monthly doses of Dihydroartemisinin-piperaquine ; IPTp=Intermittent Preventive Treatment; SP3=three doses of Sulfadoxine-Pyrimethamine; $SP_{monthly}$= monthly doses of Sulfadoxine-Pyrimethamine

Appendix 5: Decision tree of maternal anaemia

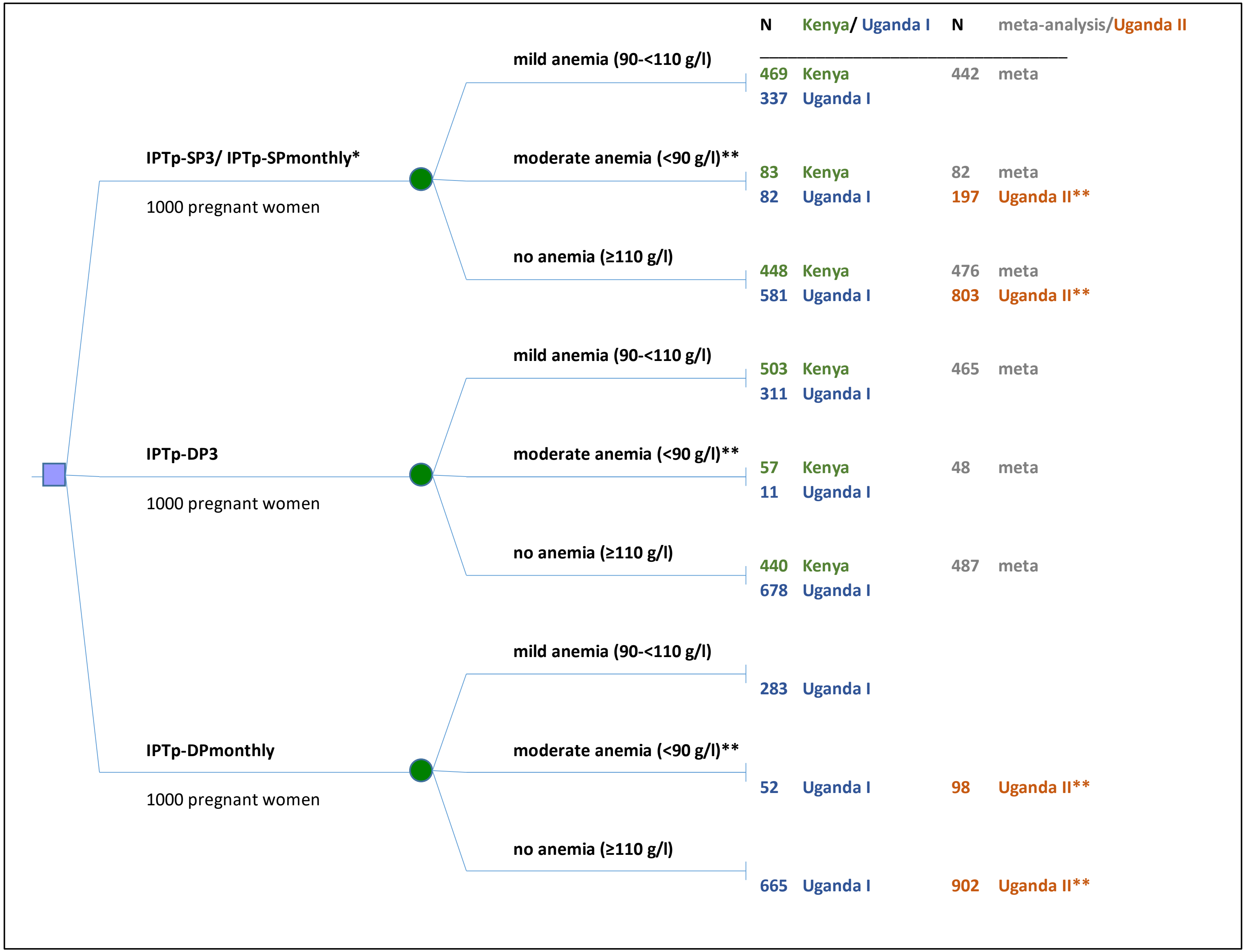

*comparison arms were: Kenya: IPTp-DP3 vs IPTp-SP3; Uganda I: IPTp-DPmonthly, IPTp-DP3 vs IPTp-SP3; Uganda II: IPTp-DPmonthly vs IPT-SPmonthly and meta: IPTp-DP3 vs IPTp-SP3; for an overview see appendix 1.

** for Uganda II only data for moderate anaemia were available, which was defined as Hemoglobin of <100g/l

Appendix 6: Decision tree of maternal clinical malaria

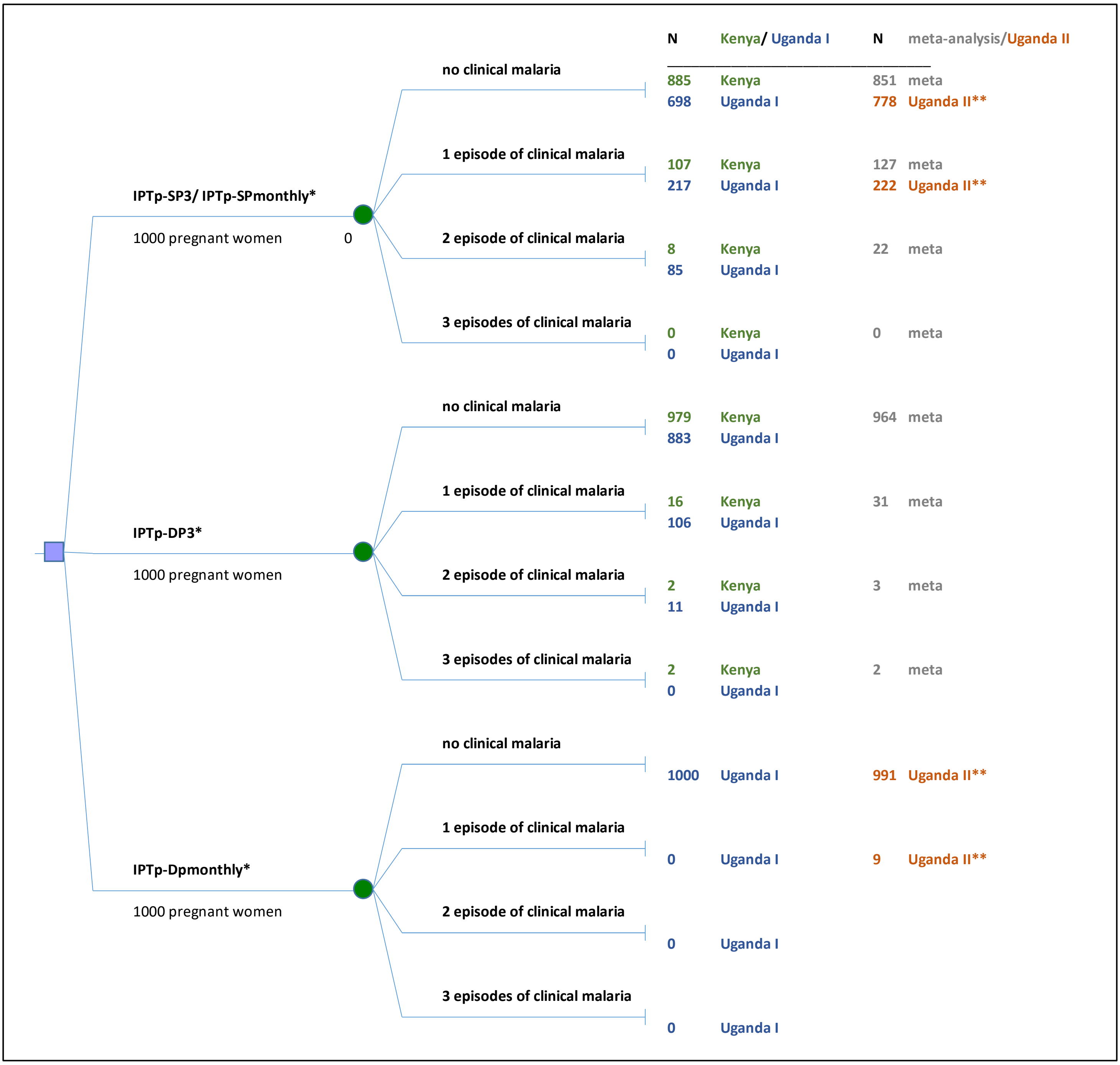

*comparison arms were: Kenya: IPTp-DP3 vs IPTp-SP3; Uganda I: IPTp-DPmonthly, IPTp-DP3 vs IPTp-SP3; Uganda II: IPTp-DPmonthly vs IPT-SPmonthly and meta: IPTp-DP3 vs IPTp-SP3; for an overview see appendix 1.

** for Uganda II only total number of episodes of malaria were available

Appendix 7: Meta-analysis results child outcomes

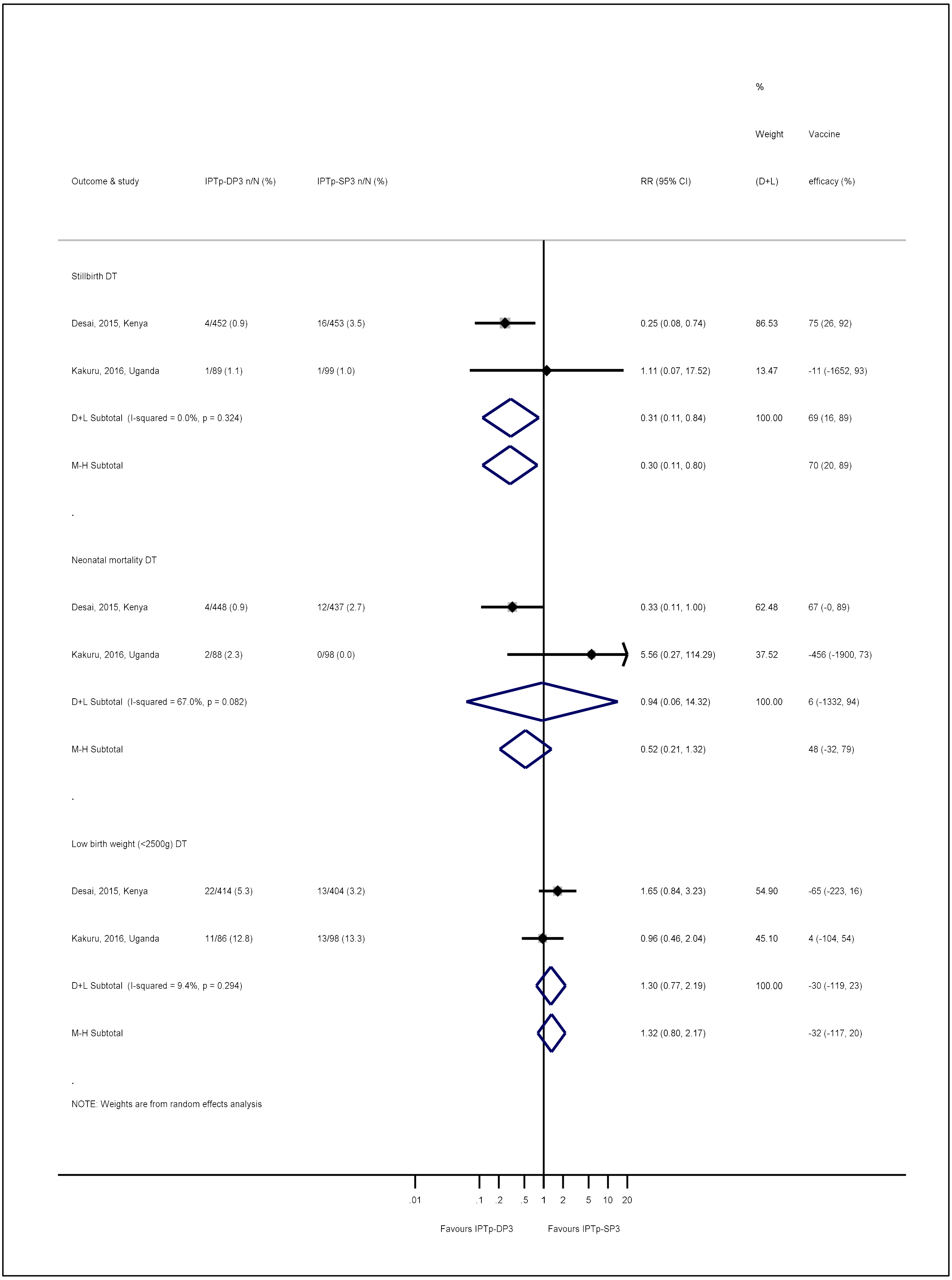

Appendix 7: Meta-analysis results for child outcomes comparing IPTp-DP3 with IPTp-SP3 using results from Desai, et al., 2015[1] and Kakuru, et al., 2016[2], following the structure of the decision tree. The meta-analysis was conducted using fixed and random effects models with a significance level of 0.05. Meta-analysis results for maternal outcomes

can be found in appendix 8. “D+L Subtotal” reports random effects results and “M-H Subtotal” fixed effect results. Diamonds represent summary effect of studies. P-values following the I^2 statistics represent the χ^2 test for heterogeneity.

Abbreviations: DP3=3 doses of Dihydroartemisinin-piperaquine; DT=Decision tree; IPTp=Intermittent Preventive Treatment; SP3=three doses of Sulfadoxine-Pyrimethamine; 95% CI=95% Confidence interval

Appendix 8: Meta-analysis results maternal outcomes

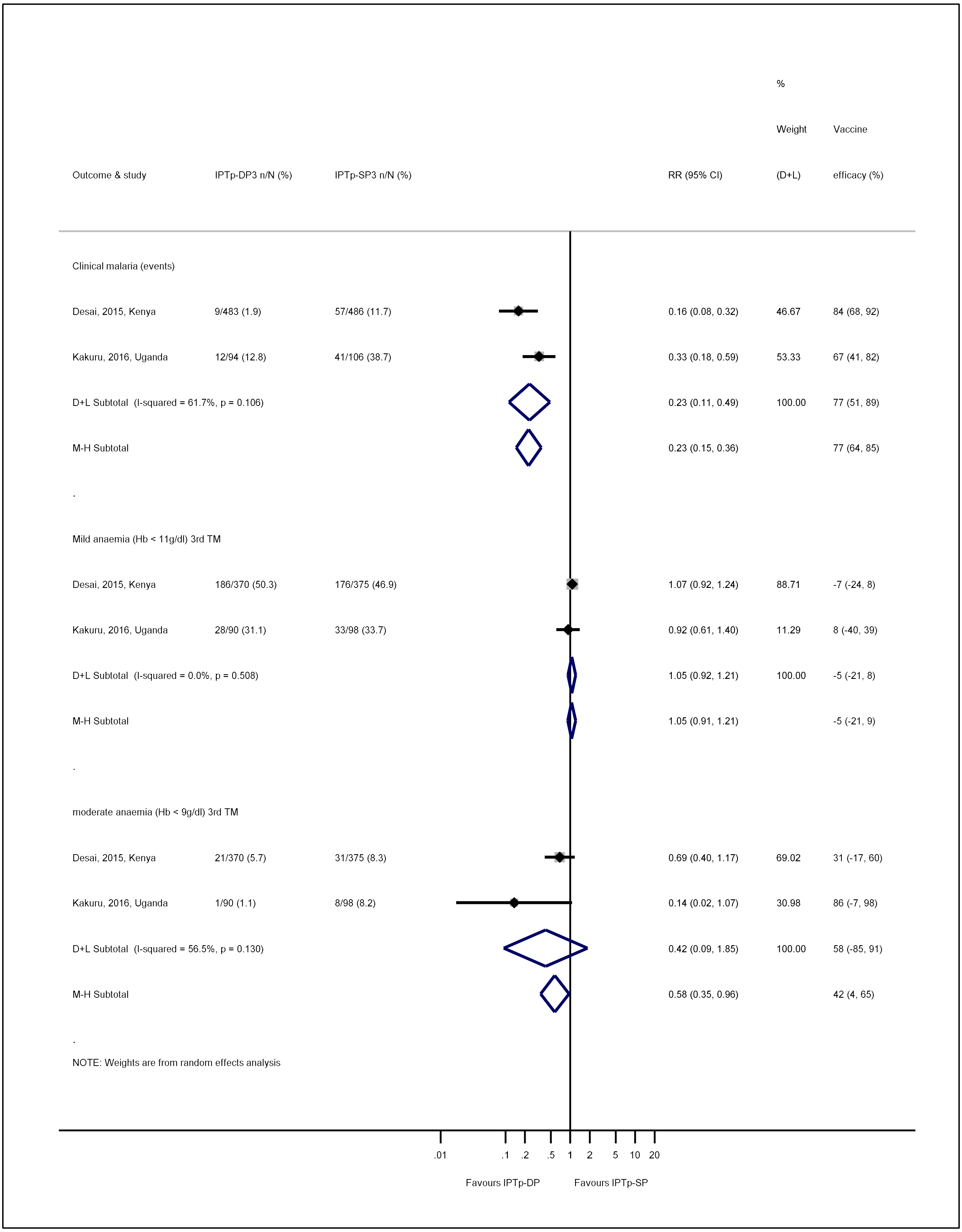

Appendix 8: Meta-analysis results for maternal outcomes using results from Desai, et al., 2015[1] and Kakuru, et al., 2016 [2], following the structure of the decision tree. The primary meta-analysis was conducted using random effects with a significance level of 0.05. "D+L Subtotal" reports random effect results and "M-H Subtotal" fixed effect results. Diamonds represent summary effect of studies. P-values following the I^2 statistics represent the χ^2 test for heterogeneity

Abbreviations: DP3=3 doses of Dihydroartemisinin-piperaquine; DT=Decision tree; IPTp=Intermittent Preventive Treatment; SP3=three doses of Sulfadoxine-Pyrimethamine; 95% CI=95% Confidence interval

Appendix 9: Cost effectiveness planes Uganda-I data, IPTp-DP3 versus IPTp-SP3

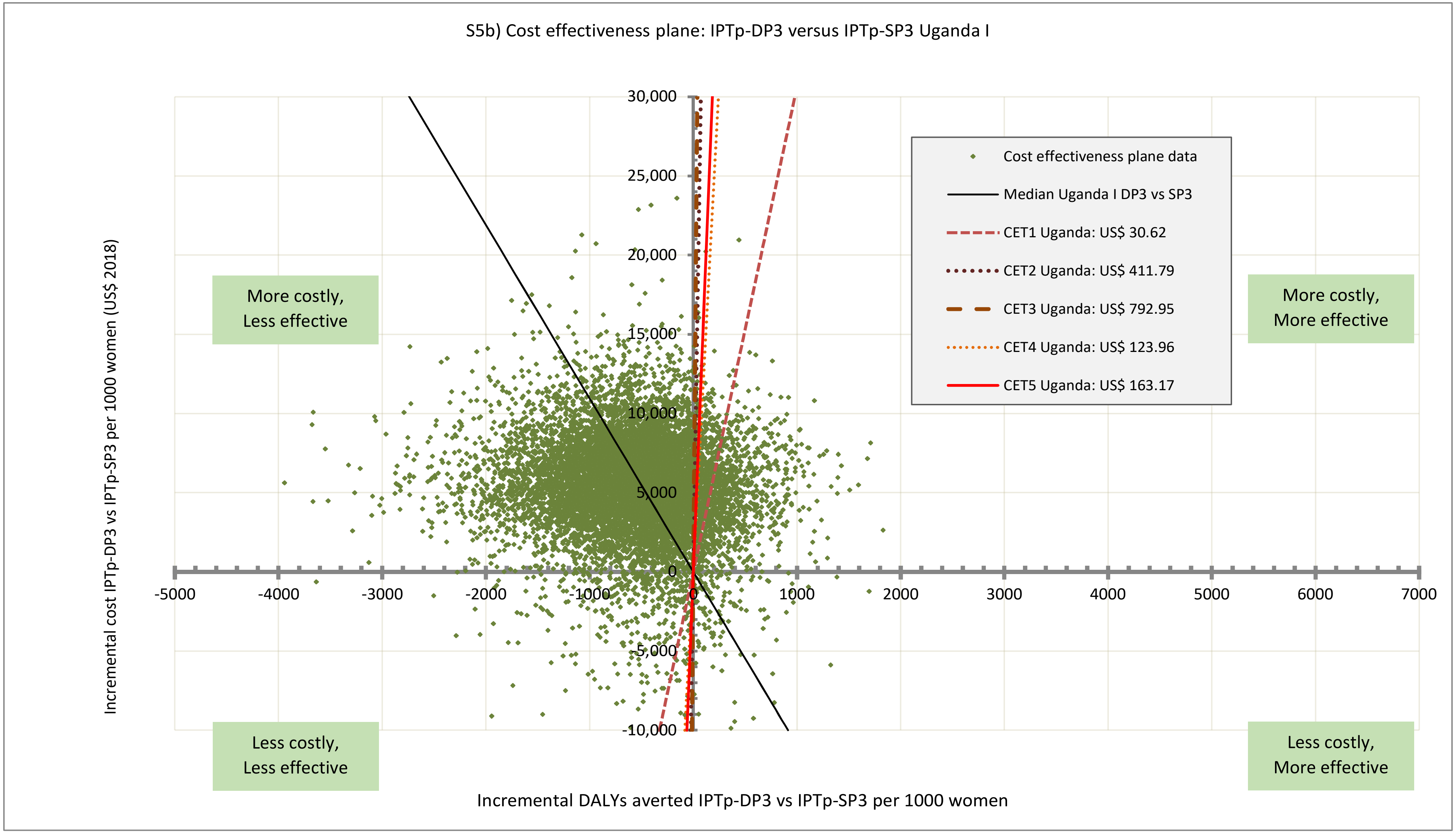

Appendix 10: Cost effectiveness planes Uganda-I data, IPTp-DP$_{monthly}$ versus IPTp-DP3

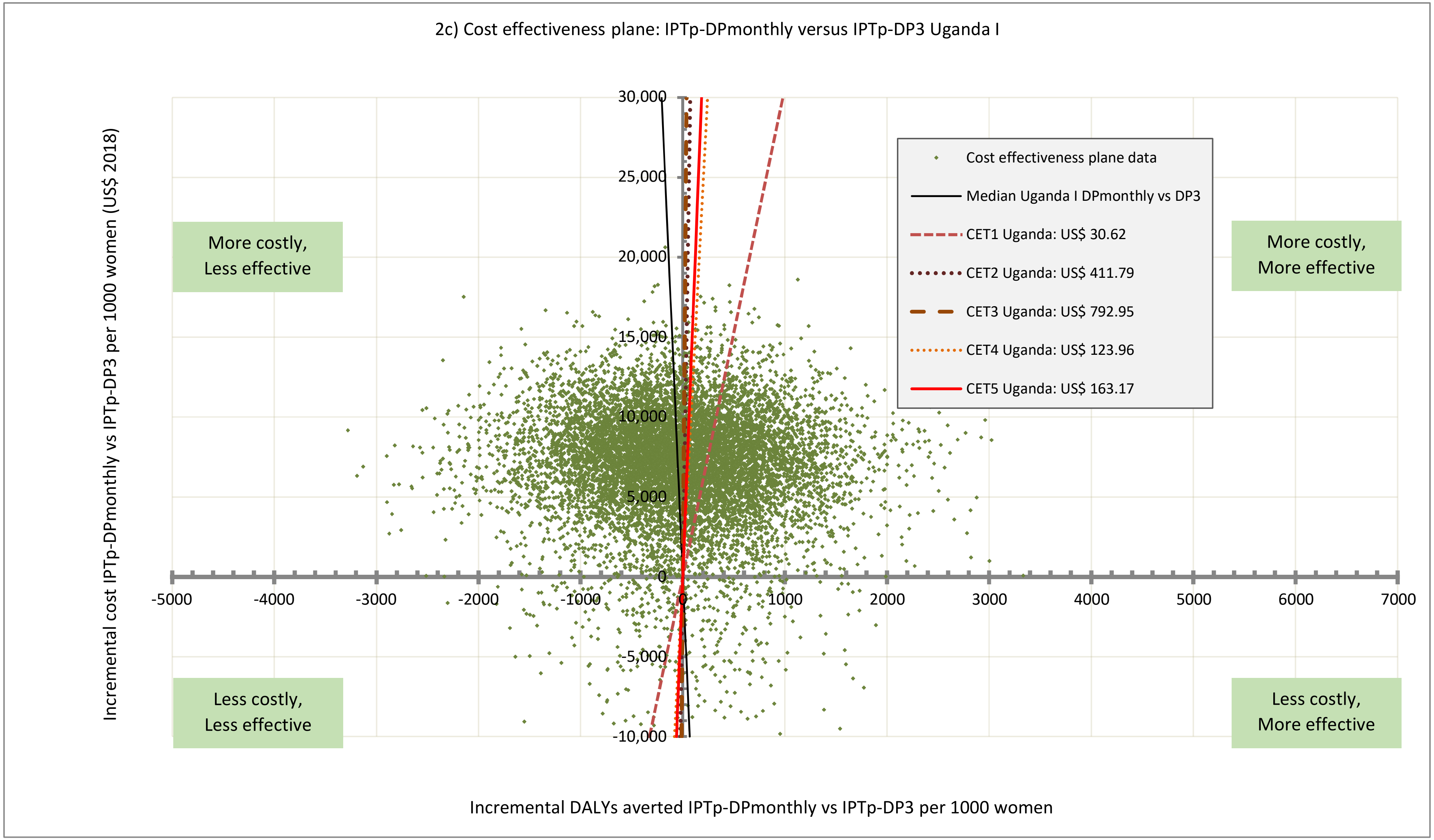

Appendix 11: Cost effectiveness planes Uganda-I data, IPTp-DP$_{monthly}$ versus IPTp-SP3

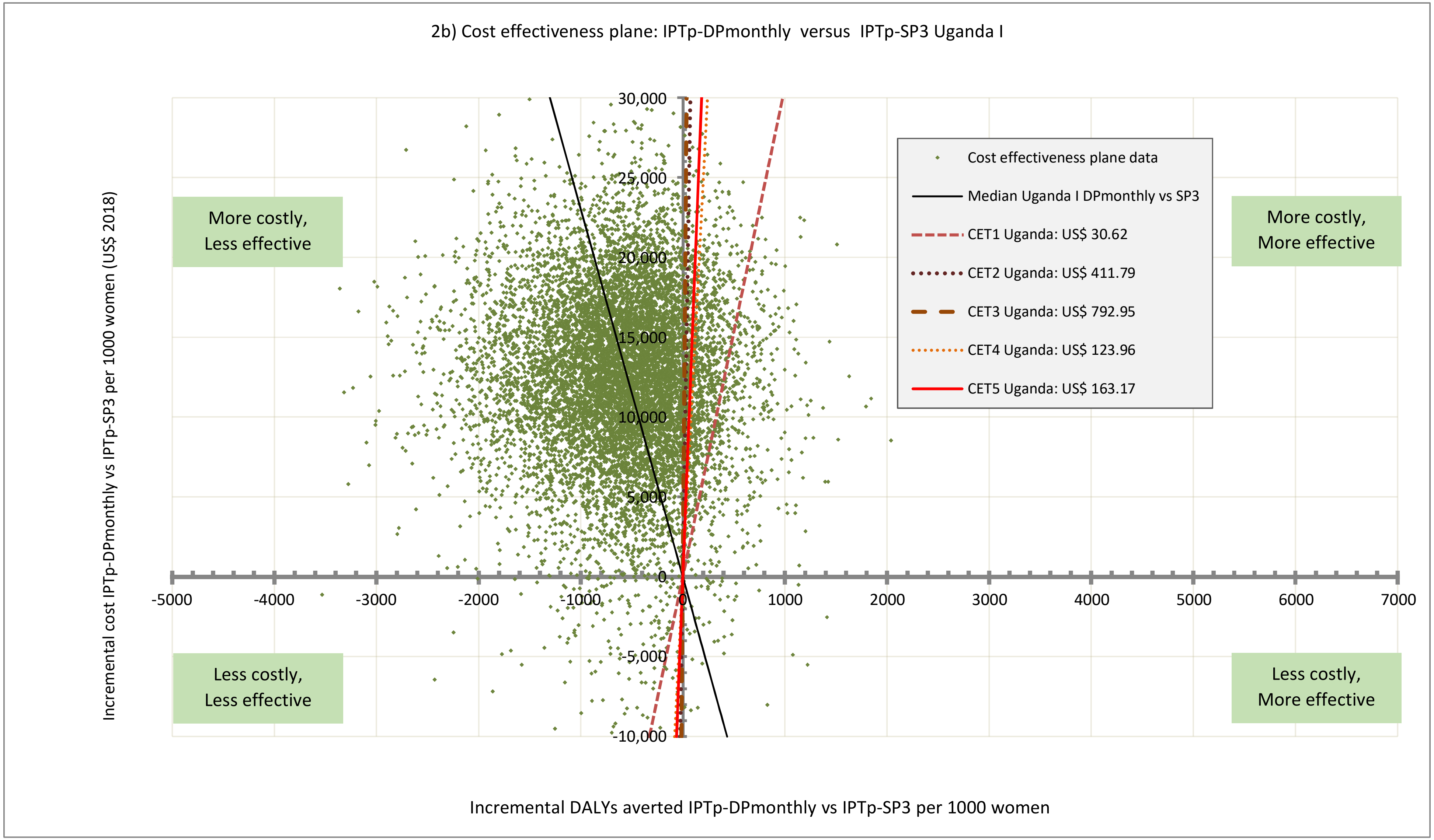

Appendix 12: Cost effectiveness planes Kenya data, IPTp-DP3 versus IPTp-SP3

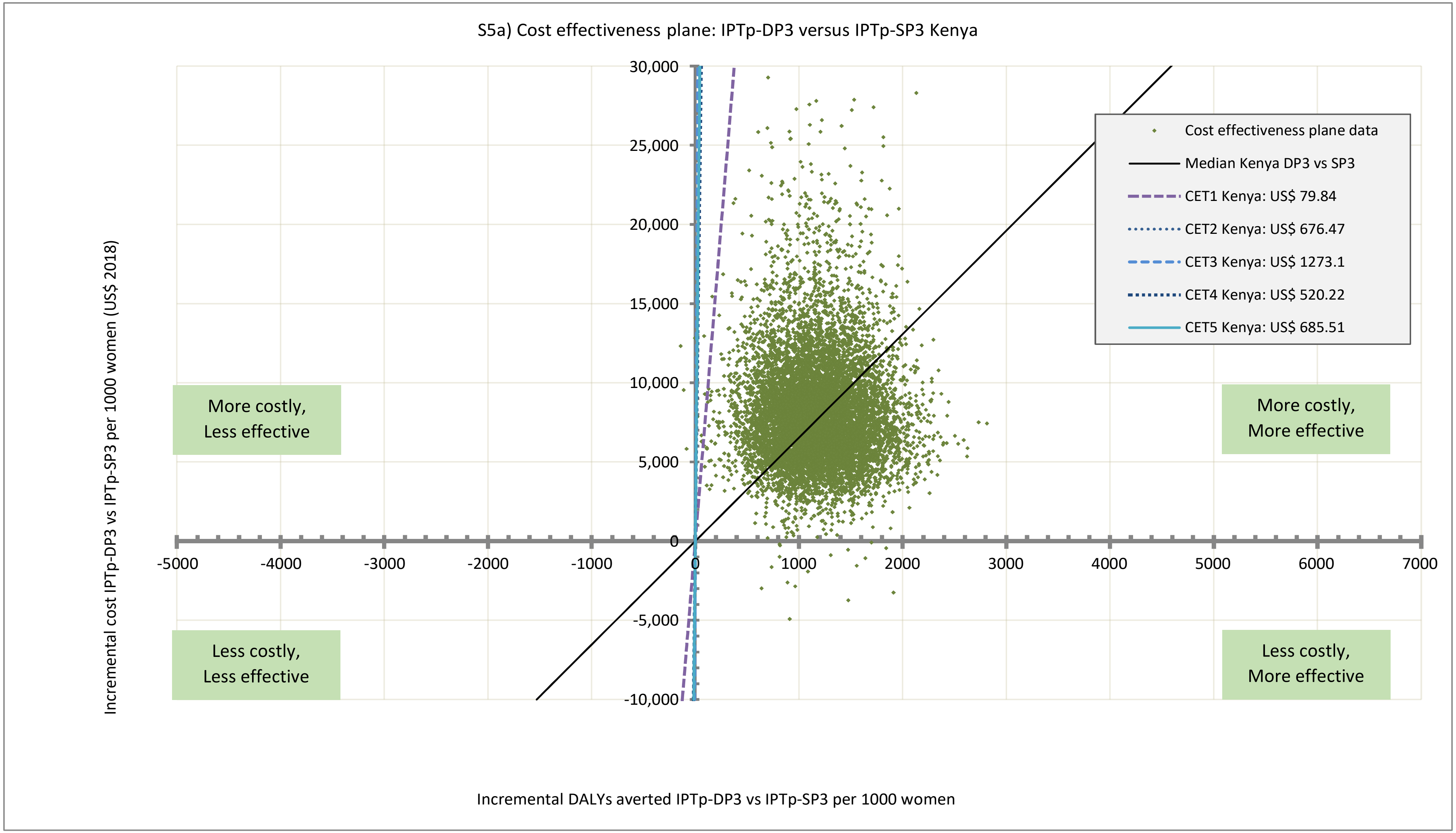

Appendix 9-12: Cost-effectiveness planes for a hypothetical cohort of 1000 pregnant women comparing:
Appendix 9:IPTp-DP3 versus IPTp-SP3 using Uganda-I results [2]
Appendix 10: IPTp-$DP_{monthly}$ versus IPTp-DP3 using Uganda-I results [2]
Appendix 11: IPTp-$DP_{monthly}$ versus IPTp-SP3 using Uganda-I results [2]
Appendix 12: IPTp-DP3 versus IPTp-SP3 using Kenya results [1]
The cost-effectiveness planes display the results of the probabilistic sensitivity analysis (PSA) using 10,000 simulations. On the X-axis, the incremental DALYs averted are shown per 1000 pregnant women and on the Y-axis the incremental costs per 1000 pregnant women. Each green dot represents one simulation, where the value for each parameter is sampled from a distribution (see table 1 for input parameters). In addition, the median of the simulations, as well as five country-specific cost-effectiveness thresholds (CET 1-5) are shown. The CETs were estimated using country-specific CETs published by Woods, et al., 2016 [4] and Ochalek et al., 2018 [5], adjusted for inflation. These were for Kenya US$ 79.8 (CET1), US$ 676.5 (CET2), US$ 1273.1 (CET3), US$ 520.2 (CET4) and US$ 685.5 (CET5) & for Uganda US$ 30.6 (CET1), US$ 411.8 (CET2), US$ 793.0 (CET3), US$ 124.0 (CET4) and US$ 163.2 (CET5).
Abbreviations: CET=Cost-effectiveness threshold; DALY=Disablity adjusted life year; DP3=3 doses of Dihydroartemisinin-piperaquine; $DP_{monthly}$=monthly doses of Dihydroartemisinin-piperaquine from 2nd trimester onwards; IPTp=Intermittent Preventive Treatment; PSA=Probabilistic sensitivity analysis; SP3=three doses of Sulfadoxine-Pyrimethamine; $SP_{monthly}$=monthly doses of Sulfadoxine-Pyrimethamine

Appendix 13: Deterministic sensitivity analysis: Tornado diagram of IPTp-DP$_{monthly}$ versus DP3 using Uganda-I data

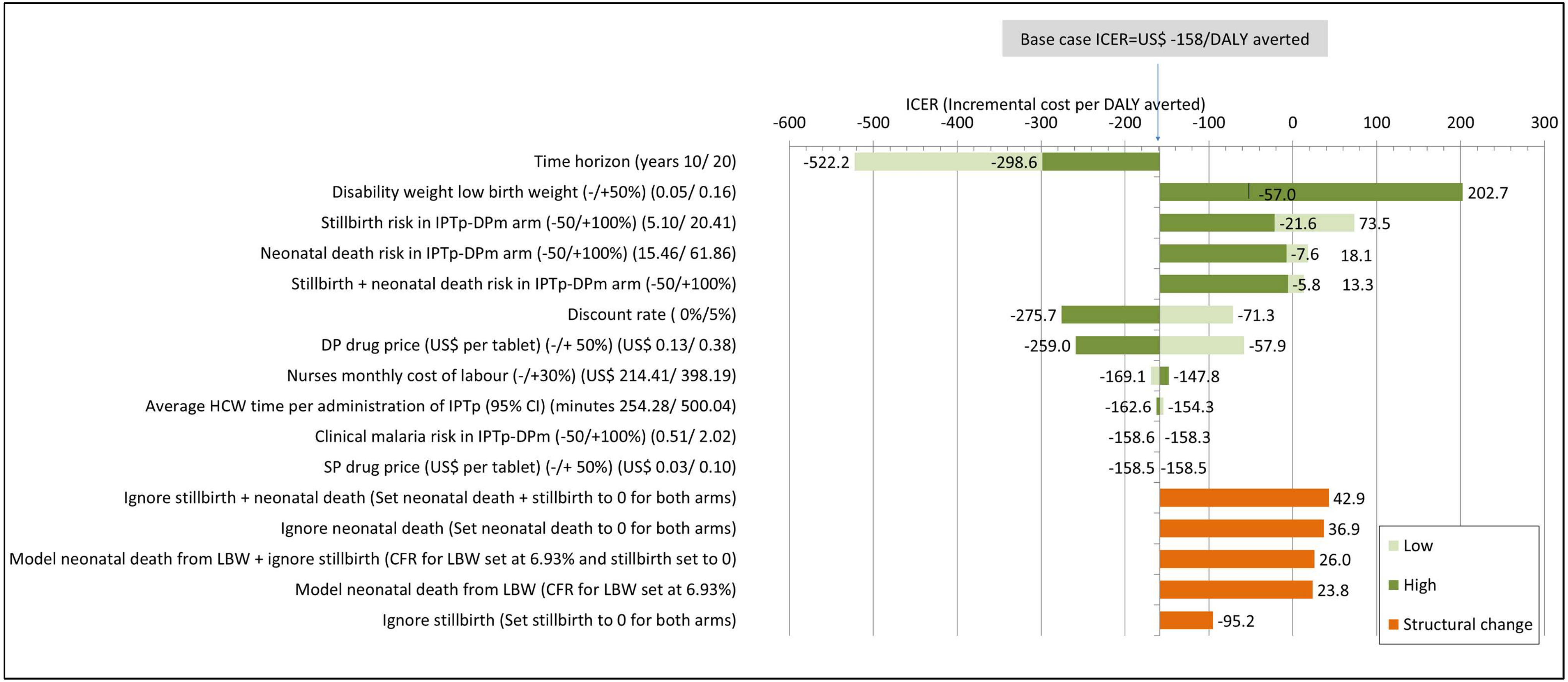

Appendix 13: Deterministic sensitivity analysis using Tornado diagrams comparing IPTp-DP$_{monthly}$ versus IPTp-DP3 using Uganda-I results [2]. The Base case ICER (US$ -158/DALY averted) is shown with a blue arrow in the graph. Eleven single parameters that were varied individually using lower and higher estimates are shown in green, alongside five more structural changes to the model which address the fact that the outcomes driving the cost-effectiveness vary among the three trials, shown in red.

Abbreviations: DALY=Disability-adjusted life year; DP3=3 doses of Dihydroartemisinin-piperaquine; DP$_{monthly}$=monthly doses of Dihydroartemisinin-piperaquine from 2nd trimester onwards; ICER=Incremental cost-effectiveness ratio; IPTp=Intermittent Preventive Treatment; LBW=Low birth weight; SP3=three doses of Sulfadoxine-Pyrimethamine; SP$_{monthly}$=monthly doses of Sulfadoxine-Pyrimethamine;

Appendix 14: Deterministic sensitivity analysis: Tornado diagram of IPTp-DP$_{monthly}$ versus SP$_{monthly}$ using Uganda-II data

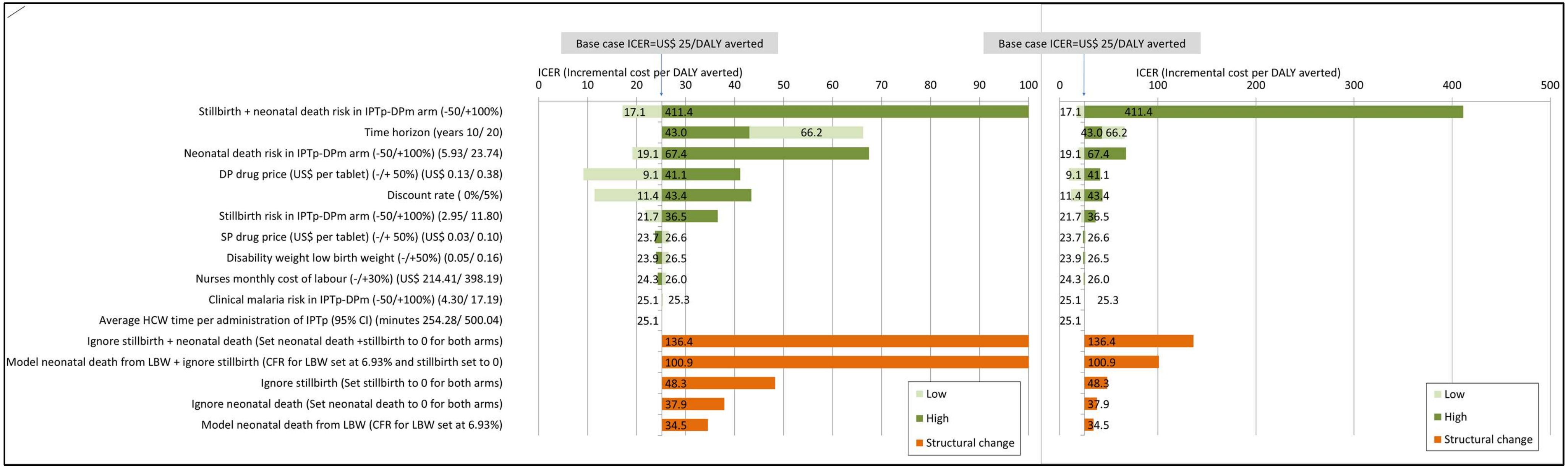

Appendix 14: Deterministic sensitivity analyses using Tornado diagrams comparing IPTp-DP$_{monthly}$ versus IPTp-SP$_{monthly}$ using Uganda-II results [3]. The Base case ICER (US$25/DALY averted) is shown with a blue arrow in the graph. Eleven single parameters that were varied individually using lower and higher estimates are shown in green, alongside five more structural changes to the model which address the fact that the outcomes driving the cost-effectiveness vary among the three trials, shown in red.

Abbreviations: DALY=Disability-adjusted life year; DP3=3 doses of Dihydroartemisinin-piperaquine; DP$_{monthly}$=monthly doses of Dihydroartemisinin-piperaquine from 2nd trimester onwards; ICER=Incremental cost-effectiveness ratio; IPTp=Intermittent Preventive Treatment; LBW=Low birth weight; SP3=three doses of Sulfadoxine-Pyrimethamine; SP$_{monthly}$=monthly doses of Sulfadoxine-Pyrimethamine;

References

1. Desai M, Gutman J, L'Lanziva A, et al. Intermittent screening and treatment or intermittent preventive treatment with dihydroartemisinin-piperaquine versus intermittent preventive treatment with sulfadoxine-pyrimethamine for the control of malaria during pregnancy in western Kenya: an open-label, three-group, randomised controlled superiority trial. *Lancet* 2015; **386**(10012): 2507-19.
2. Kakuru A, Jagannathan P, Muhindo MK, et al. Dihydroartemisinin-Piperaquine for the Prevention of Malaria in Pregnancy. *The New England journal of medicine* 2016; **374**(10): 928-39.
3. Kajubi R, Ochieng T, Kakuru A, et al. Monthly sulfadoxine-pyrimethamine versus dihydroartemisinin-piperaquine for intermittent preventive treatment of malaria in pregnancy: a double-blind, randomised, controlled, superiority trial. *Lancet* 2019; **393**(10179): 1428-39.
4. Woods B, Revill P, Sculpher M, Claxton K. Country-Level Cost-Effectiveness Thresholds: Initial Estimates and the Need for Further Research. *Value Health* 2016; **19**(8): 929-35.
5. Ochalek J, Lomas J, Claxton K. Estimating health opportunity costs in low-income and middle-income countries: a novel approach and evidence from cross-country data. *BMJ global health* 2018; **3**(6): e000964.
